# Astroglial-targeted expression of the fragile X CGG repeat premutation in mice yields RAN translation, motor deficits and possible evidence for cell-to-cell propagation of FXTAS pathology

**DOI:** 10.1186/s40478-019-0677-7

**Published:** 2019-02-26

**Authors:** H. Jürgen Wenzel, Karl D. Murray, Saif N. Haify, Michael R. Hunsaker, Jared J. Schwartzer, Kyoungmi Kim, Albert R. La Spada, Bryce L. Sopher, Paul J. Hagerman, Christopher Raske, Lies-Anne W.F.M. Severijnen, Rob Willemsen, Renate K. Hukema, Robert F. Berman

**Affiliations:** 1 Department of Neurological Surgery, University of California, Davis, Davis, CA USA; 2 Department of Psychiatry and Behavioral Sciences, University of California, Davis, Davis, CA USA; 3 Graduate Program in Neuroscience, University of California, Davis, Davis, CA USA; 4 Department of Clinical Genetics, Erasmus MC, Rotterdam, The Netherlands; 5 Department of Biochemistry and Molecular Medicine, University of California, Davis, Davis, CA USA; 6 Division of Biostatistics, Department of Public Health Sciences, University California Davis, Davis, CA USA; 7 Departments of Neurology, Neurobiology, and Cell Biology, and the Duke Center for Neurodegeneration & Neurotherapeutics, Duke University School of Medicine, Durham, NC USA; 8 Department of Neurology, University of Washington School of Medicine, Seattle, WA USA; 9 Program in Neuroscience and Behavior, Department of Psychology and Education, Mount Holyoke College, South Hadley, MA USA

**Keywords:** FXTAS, Fragile X premutation, Mouse model, Neurodegeneration, Glia, RAN translation, FMRpolyG, Non-cell-autonomous, Electron microscopy of inclusions

## Abstract

The fragile X premutation is a CGG trinucleotide repeat expansion between 55 and 200 repeats in the 5′-untranslated region of the fragile X mental retardation 1 (*FMR1*) gene. Human carriers of the premutation allele are at risk of developing the late-onset neurodegenerative disorder, fragile X-associated tremor/ataxia syndrome (FXTAS). Characteristic neuropathology associated with FXTAS includes intranuclear inclusions in neurons and astroglia. Previous studies recapitulated these histopathological features in neurons in a knock-in mouse model, but without significant astroglial pathology. To determine the role of astroglia in FXTAS, we generated a transgenic mouse line (Gfa2-CGG99-eGFP) that selectively expresses a 99-CGG repeat expansion linked to an enhanced green fluorescent protein (eGFP) reporter in astroglia throughout the brain, including cerebellar Bergmann glia. Behaviorally these mice displayed impaired motor performance on the ladder-rung test, but paradoxically better performance on the rotarod. Immunocytochemical analysis revealed that CGG99-eGFP co-localized with GFAP and S-100ß, but not with NeuN, Iba1, or MBP, indicating that CGG99-eGFP expression is specific to astroglia. Ubiquitin-positive intranuclear inclusions were found in eGFP-expressing glia throughout the brain. In addition, intracytoplasmic ubiquitin-positive inclusions were found outside the nucleus in distal astrocyte processes. Intriguingly, intranuclear inclusions, in the absence of eGFP mRNA and eGFP fluorescence, were present in neurons of the hypothalamus and neocortex. Furthermore, intranuclear inclusions in both neurons and astrocytes displayed immunofluorescent labeling for the polyglycine peptide FMRpolyG, implicating FMRpolyG in the pathology found in Gfa2-CGG99 mice. Considered together, these results show that Gfa2-CGG99 expression in mice is sufficient to induce key features of FXTAS pathology, including formation of intranuclear inclusions, translation of FMRpolyG, and deficits in motor function.

**Electronic supplementary material:**

The online version of this article (10.1186/s40478-019-0677-7) contains supplementary material, which is available to authorized users.

## Introduction

The fragile X premutation is defined as an expanded (CGG)_n_ trinucleotide repeat in the 5′-untranslated region of the *FMR1* gene. Clinical and genetic studies of patients have indicated that carriers of the premutation allele, defined as a repeat length between 55 and 200 CGGs, are at risk of developing the late-onset neurodegenerative disorder, fragile X-associated tremor/ataxia syndrome (FXTAS) [27, 28, 35]. Elevated *FMR1* mRNA levels found in cells of premutation carriers support the concept of a “toxic” *mRNA* gain-of-function mechanism of pathophysiology in FXTAS [25], likely via sequestration of RNA-binding proteins by expanded CGG repeat-containing RNA [48]. Repeat-associated non-ATG translation (RAN) of a toxic in polyglycine-containing peptide, FMRpolyG, from the expanded-repeat mRNA may also contribute to FXTAS pathology [36, 47]. The principal clinical symptoms of FXTAS include progressive intention tremor and ataxia, peripheral neuropathy, neuropsychological involvement (anxiety, depression), and cognitive impairments and dementia at late stages of the disorder [4, 25, 26]. Radiologic changes observed by MRI include increased T2 signal (hyperintensities) in cerebral white matter and in the middle cerebellar peduncle (the “MCP sign”), as well as global brain atrophy [8]. Levels of *FMR1* mRNA are elevated and levels of FMRP are slightly decreased in FXTAS. The neuropathological hallmark of FXTAS is the presence of spherical eosinophilic intranuclear inclusions in neurons and astroglia throughout the brain that are immunoreactive for ubiquitin [23, 24, 51].

The CGG KI mouse model of FXTAS shows similar neurobehavioral features that appear to be similar to those in FXTAS [20]. These include gait ataxia and visuomotor deficits in the ladder-rung [31] and rotarod tests [54], anxiety in the open field [10] and cognitive impairment [30, 32]. They also show ubiquitin-positive spherical inclusions in neurons and astrocytes similar to those found in FXTAS brains [6, 56]. The inclusions are found throughout the brain in all neocortical regions, hippocampus, hypothalamus, brain stem nuclei (e.g., reticular formation, inferior olivary and dentate nuclei) and in Bergmann glia in cerebellum [56, 59]. The topographical distribution and frequency of intranuclear inclusions increase with age and length of the CGG repeat segment, and also vary between brain regions [56, 59]. Pathology in the CGG KI mouse model differs from FXTAS pathology by the absence of tremors and the relatively few numbers of astrocytes with intranuclear inclusions [2]. Ubiquitin-positive inclusions were never observed in neurons or astroglia of WT mice in any brain region at any age [46].

To determine if expression of a CGG trinucleotide repeat expansion in astroglia is sufficient to induce pathology in astroglia, and to characterize the role of astroglia in FXTAS, we created a transgenic mouse line (Gfa2-CGG99-eGFP) that expresses a 99 CGG repeat expansion in astrocytes throughout the brain and in Bergmann glia in the cerebellum. Expression is driven by an astroglia-specific Gfa2 promoter fused to an eGFP reporter gene. In these mice, immunocytochemical analysis of eGFP expression patterns revealed that CGG99-eGFP expression co-localized with astroglia markers, but not with neuronal, microglia, or oligodendroglia markers, indicating that CGG99-eGFP expression was specific for astroglia and Bergmann glia. Double-immunostaining for ubiquitin revealed the presence of intranuclear inclusions in eGFP-positive glia throughout the brain, as well as ubiquitin-positive inclusions in the cytoplasm of astrocyte processes. Surprisingly, we also observed intranuclear inclusions in NeuN-positive neurons of the hypothalamus and neocortex, though these cells did not express the CGG99-eGFP transcript. The presence of cytoplasmic inclusions in astrocytes, ectopic inclusions and inclusions in neurons suggests a spread of pathology from astrocytes to neurons by as yet unknown mechanisms. Both glial and neuronal inclusions stained positive for the RAN translation product FMRpolyG [9, 52]. These results indicate that an expanded CGG-99 repeat in astroglia is sufficient to induce formation of ubiquitin- and FMRpolyG-positive intranuclear inclusions - key features of FXTAS pathology, and that the Gfa2-CGG99-eGFP mouse will be a valuable model to delineate neuron-astroglia interactions that contribute to FXTAS disease pathogenesis.

## Materials and methods

### Generation of *Gfa2*-CGG99-eGFP and *Gfa2*-CGG11-eGFP mice

**Fig. 1:**
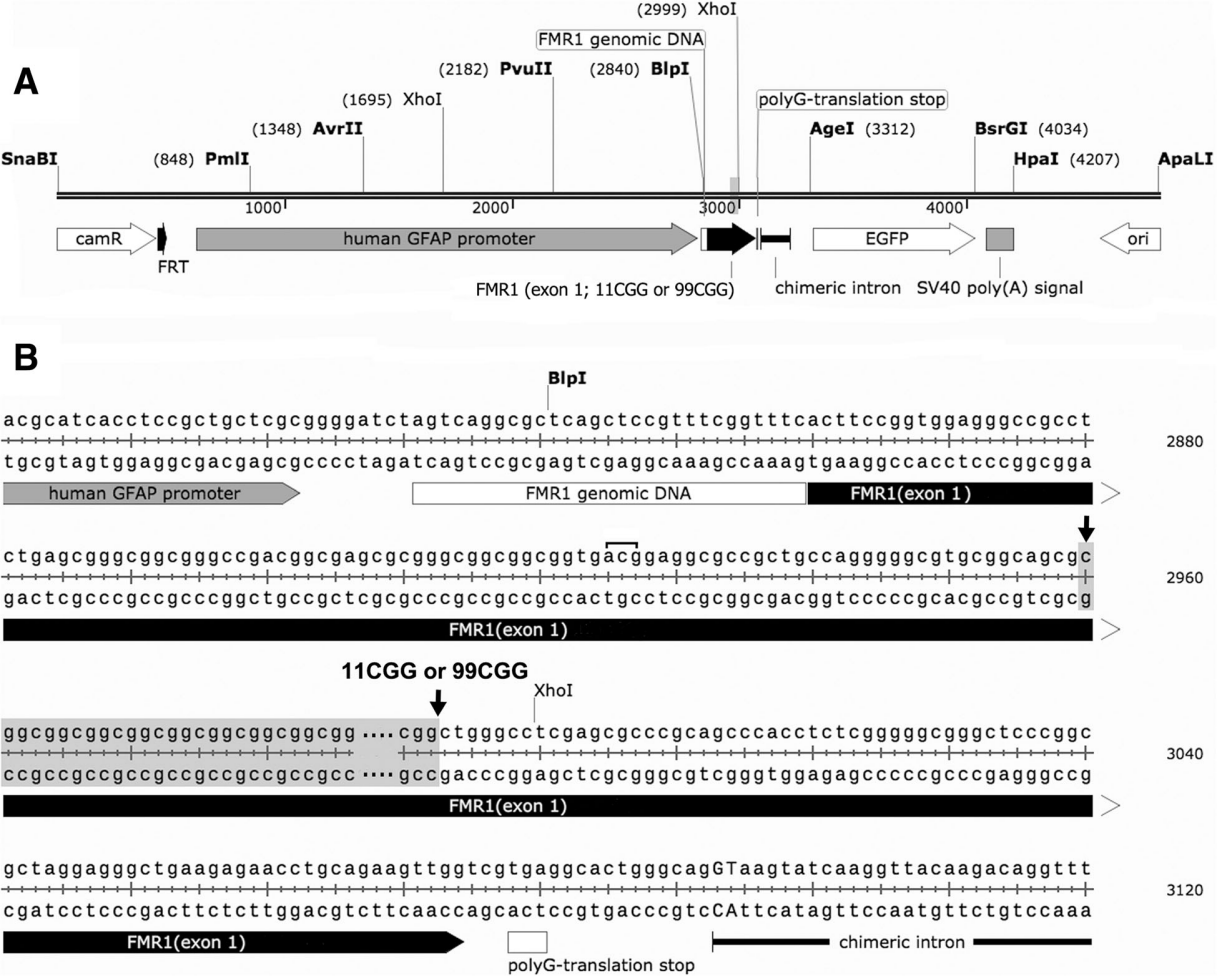
**a** Diagram of DNA fragment used for pronuclear injection with either an 11CGG or 99CGG trinucleotide repeat expansion on exon 1. **b** Nucleotide sequence of DNA construct used to make the GFAP-CGG11-EGFP or GFAP-CGG99-EGFP transgenic mice. The sequence contained either an 11CGG or 99CGG trinucleotide repeat sequence for the two transgenic mouse lines that was located between the two arrows in **b**. The bracketed acg sequence upstream of the repeat sequence shows the alternative translation start site supporting repeat-associated non-ATG (RAN) translation as described in Sellier, et al., 2017

Transgenic mice on a C57BL/6j background were generated with an expanded CGG99 trinucleotide repeat segment (Gfa2-CGG99-eGFP) or a typical mouse-sized CGG11 (Gfa2-CGG11-eGFP) repeat sequence. For simplicity, the Gfa2-CGG99-eGFP mice are referred to as Gfa2-CGG99 and the Gfa2-CGG11-eGFP mice as Gfa2-CGG11 control mice, respectively. Wildtype (WT) non-transgenic littermates were generated from breeding the Gfa2-CGG99 transgenic mice with WT C57BL/6j mice. These WT littermates mice were used as controls for behavioral studies to avoid litter effects and because sufficient numbers of the Gfa2-CGG11 transgenic mice were not available. Genotype was verified in all mice by PCR from tail-snips. A diagram of the DNA constructs and the nucleotide sequences used for pronuclear injection are shown in Fig. 1a and b, respectively. Expression vector maps are included in Additional file 1: Figure S1. Expression was restricted to astrocytes and Bergmann glia using the astrocyte-specific *Gfa2* promoter, with the enhanced green fluorescent protein (eGFP) reporter used to identify cells expressing the Gfa2-CGG99-eGFP or the normal length Gfa2-CGG11 transgene. The *eGFP* sequence was derived from the pBR-eGFP vector. Expression of the CGG99 and CGG11 trinucleotide repeat expansions and eGFP reporter are driven by ~ 2-kb of the human Gfa2 promoter, which drives astrocyte-specific expression in transgenic mice [5]. cDNA derived from patient and control peripheral blood lymphocytes was used to isolate ~ 226 bp of *FMR1* 5′-UTR sequence as well as the CGG repeats, which were cloned into Blp I and Pst I restriction sites. The construct also contains a chimeric intron upstream of eGFP to enhance expression levels. Gfa2-CGG11-eGFP and Gfa2-CGG99-eGFP clones (i.e., clones 3 and 11, respectively) were digested with ApaLI and SnaBI (10 μg each) and the respective 4.8 and 5.1 kb restriction fragments were purified away from the 0.9 kb vector backbone on an agarose gel. The purified Gfa2-CGG11-eGFP (86 ng/ul & 1.8260/280 ratio) and Gfa2-CGG99-eGFP (62 ng/ul & 1.8260/280 ratio) DNA fragments were then microinjected into pronuclei of oocytes from C57BL/6J x C3H/HeJ F1 hybrids at the University of Washington Microinjection Service Laboratory. Six of the 26 Gfa2-CGG11-eGFP mice screened (LS-2997, LS-3006, LS-3009, LS-3011, LS-3025, LS-3030 were found to be positive for the transgene by PCR. Six of the 27 Gfa2-CGG99-EGFP mice screened (LS-3046, LS-3049, LS-3060, LS-3061, LS-3065, LS-3072) were also found to be positive for the transgene by PCR. The Gfa2-CGG11 and Gfa2-CGG99 mouse lines used in these experiments were selected based on matched, high expression levels by TaqMan real time PCR using primers and probes to the eGFP gene as follows: forward, 5′- GTC CGC CCT GAG CAA AGA -3′; reverse, 5′- TCC AGC AGG ACC ATG TGA TC -3′; Fam-probe, 5′- CCC AAC GAG AAG CG -3′.

Eight male Gfa2-CGG99 mice and five male Gfa2-CGG11 control mice between 4 and 16 months old were used for histological and molecular studies. In addition, brains of two male CGG_n_ knock-in (CGG128 and CGG159) and 2 WT mice 16 months of age were immunostained for ubiquitin-positive intranuclear inclusions in neurons and astroglia for comparison with Gfa2-CGG99 mice and Gfa2-CGG11 mice, as previously described [56]. Transgenic and WT mice were housed under conditions of constant temperature and a 12 / 12 h light-dark cycle, and with food and water ad libitum. These experiments followed the “Principles of laboratory animal care” (NIH publication No. 86–23, revised 1985), and were approved by the UC Davis Institutional Animal Care and Use Committee.

### Genotyping

DNA was extracted from mouse tails by incubating with 10 mg/ml Proteinase K (Roche Diagnostics) in 300 μl lysis buffer containing 50 mM Tris-HCl, pH 7.5, 10 mM EDTA, 150 mM NaCl, 1% SDS overnight at 55 °C. One hundred μl saturated NaCl was then added and the suspension was centrifuged. One volume of 100% ethanol was added, gently mixed, and the DNA was pelleted by centrifugation and the supernatant discarded. The DNA was washed and centrifuged in 500 μl 70% ethanol. The DNA was then dissolved in 100 μl milliQ-H_2_0. CGG repeat lengths were determined by PCR using the Expanded High Fidelity Plus PCR System (Roche Diagnostics). Briefly, approximately 500–700 ng of DNA was added to 50 μl of PCR mixture containing 2.0 μM of each primer, 250 μM of each dNTP (Invitrogen), 2% DMSO (Sigma), 2.5 M Betaine (Sigma), 5 U Expand HF buffer with Mg (7.5 μM). For the CGG KI mice the forward primer was 5′-GCTCAGCTCCGTTTCGGTTTCACTTCCGGT-3′ and the reverse primer was 5′-AGCCCCGCACTTCCACCACCAGCTCCTCCA-3′. For the Gfa2-CGG99 mice the forward primer was 5′- GTC CGC CCT GAG CAA AGA -3′; and the reverse primer was 5′- TCC AGC AGG ACC ATG TGA TC -3.’ PCR steps were 10 min denaturation at 95 °C, followed by 34 cycles of 1 min denaturation at 95 °C, annealing for 1 min at 65 °C, and elongation for 5 min at 75 °C to end each cycle. PCR ended with a final elongation step of 10 min at 75 °C. DNA CGG band sizes were determined by running DNA samples on a 2.5% agarose gel and staining DNA with ethidium bromide.

### Behavioral testing

The behavioral tests used in the present study for GFAP-CGG99-EGFP mice were selected to examine similar neurobehavioral deficits in FXTAS, including anxiety in the elevated plus-maze, ataxia and motor deficits in the ladder rung test, rotarod and TredScan apparatus, and cognitive loss using contextual fear conditioning [20].

#### Mice

Fifteen adult wildtype (WT) and 15 Gfa2-CGG99 adult male mice were used for behavioral testing. The WT and Gfa2-CGG99 mice were littermates derived from 12 litters with no more than 3 mice taken from any litter. The mice were between 21 and 24 weeks of age at the start of behavioral testing. WT littermates were used as controls to avoid litter effects in behavioral testing, and because sufficient numbers of Gfa2-CGG11 transgenic mice were not available for the behavioral studies. Mice were housed individually in a climate controlled vivarium under 12:12 h light dark cycles with food and water available ad libitum*.* Animals were tested in the order presented below.

#### Open-field locomotor activity

Locomotor activity was measured using an automated open-field activity arena (TruScan, Coulbourn Instr., Whitehall, PA). The apparatus (27.5 × 27.5 × 37.5 cm) detects movement by recording infrared beam brakes. The number of center entries, total time in the center and periphery of the arena, and frequency of rearing were automatically scored during a 90-min period.

#### Elevated plus maze

The maze consisted of two open arms (30 × 5 × 0.25 cm) and two closed arms (30 × 5 × 6 cm) elevated 60 cm above the floor. Mice were placed in the center of an elevated plus-maze and were allowed to freely explore the apparatus for 5 min while recorded by a video-tracking system (SMART, San Diego Instruments, San Diego, CA). Number of entries into open and closed arms, latency to first enter, and total time spent in open and closed arms were compared between WT and Gfa2-CGG99 mice.

#### Rotarod

Motor coordination and balance were evaluated using a Rotamex-5 rotarod with photocell detection (Columbus Instr., Columbus, OH). Prior to testing mice were given an initial training session where they were acclimated to the apparatus. Training consisted of a 120 s trial in which the rod rotated at a constant speed of 4 RPM. Mice that fell were immediately replaced and allowed to complete the training session. Twenty-four hr. later WT and Gfa2-CGG99 mice were placed on the rotarod at an initial speed of 4 RPM that accelerated by 1.0 RPM every 10 s. A trial was terminated when a mouse fell from the rod at which time the speed and latency to fall were recorded. The number of flips (i.e., 360 deg. rotations while grasping the rod) was also recorded. Each mouse was tested three times per day for three consecutive days. Mean performance time per day was defined as the average time the mouse remained on the rotarod across trials.

#### Gait analysis

Gait abnormalities in Gfa2-CGG99 mice were analyzed and compared to WT mice using a motor-driven transparent treadmill affixed with high-speed digital video camera and computer-assisted software (TreadScan, CleverSys, Reston,VA). To ensure that all mice were walking, and not galloping, the treadmill speed was adjusted for each mouse but never exceeded 20 cm/s. When the mouse reached a steady gate, video of the underside was recorded and analyzed by the system’s software that identified each paw individually and calculated gait variables. Variables included stride time, defined as the time between two initial paw contacts of the same paw, stance time, the period of time the paw is in contact with the treadmill, and swing time, the portion of the stride in which the paw is not in contact with the treadmill.

#### Ladder rung task

The ladder rung apparatus tested visuomotor coordination by measuring the number of foot slips made while traversing a horizontal ladder [31]. The apparatus consisted of two 28 cm tall × 65 cm long black walls separated by 10 cm. The floor was elevated 10 cm from the bottom of the walls and had 43 parallel 1 mm diameter bars separated by 1.5 cm. A video camera positioned at one end of the apparatus recorded the full length of the beam floor. This allowed the experimenter to score whether the mouse’s limbs extended below the beam floor as well as allowed the experimenter to observe the general posture of the mouse above the beam floor. All behavior was captured using a behavioral tracking system (SMART, San Diego Instruments, San Diego, CA). Mice were placed in the apparatus and allowed to freely explore the apparatus for 2 min. Mice typically explored the apparatus by walking the length of the apparatus, looking over the edge, and returning to the start position. The number of times a foot slipped through the beam floor was recorded and used as the dependent measure. Video recordings were later independently scored by two experimenters blinded to the genotype of the mice (intraclass correlation coefficient = 0.88, *p* < 0.002).

#### Fear conditioning

Mice were trained in a contextual fear conditioning apparatus (Med Associates Inc., Georgia, VT). Mice underwent an initial training period consisting of 2 trials separated by a 2 min inter-trial interval. Each trial consisted of the presence of 80 dB white noise (conditioned stimulus, CS) for 30 s which co-terminated with a 0.5 mA footshock (unconditioned stimulus, UCS) during the last 2 s. The testing for contextual and cued fear conditioning occurred 24 h later. For contextual conditioning, the mice were returned to the training chamber in the absence of the CS and UCS and measured for freezing behavior during a five-minute period. For cued fear conditioning, mice were placed in an altered context chamber modified by new floor and side inserts. Mice were measured for freezing during an initial 3-min period and during a 3 min presentation of the CS. Freezing was defined as the cessation of movement other than respiration for ≥750 milliseconds.

### Light microscopy/Electron microscopy/immunocytochemistry

#### General tissue preparation

Histological procedures were previously described in detail [56]. In brief, mice were anesthetized with sodium pentobarbital (100 mg/kg, i.p.), then perfused with isotonic saline followed by a solution of 4% buffered paraformaldehyde (PFA), and post-fixed in the same solution for 1 h at 4 °C. The brains were cryoprotected in buffered 10% sucrose for 1 h, followed by buffered 30% sucrose for 24 h at 4 °C, then rapidly frozen on dry ice. Thirty μm frozen sagittal sections were cut on a sliding microtome, and collected into a series of every fifth section in 30% sucrose. Single sets of sections were selected for further processing that included: cresyl violet and/or H&E staining for general histological evaluation, electron microscopy to analyze the ultrastructure of intranuclear inclusions in glia and neurons, immunocytochemistry/immunofluorescence for neuronal and glial cell markers, including ubiquitin staining used to visualize intranuclear inclusions. These ubiquitin-positive inclusions are the hallmark histopathology in FXTAS patients and are also found in astrocytes and neurons in CGG KI mice [23, 24, 59].

### Immunocytochemistry

Immunocytochemical and immunofluorescence techniques were used to visualize the occurrence and distribution of intranuclear inclusions, specifically in brain astroglia and cerebellar Bergmann glia of Gfa2-CGG99, of Gfa2-CGG11 control and WT mice. Subsets of alternate sections were processed for immunocytochemistry using a modification of the avidin-biotin complex (ABC)-peroxidase technique [29] as previously described [56]. Briefly, free-floating sections were rinsed in PB (pH 7.4) and pretreated with 0.1% sodium borohydride for antigen retrieval. Endogenous peroxidases were inactivated by treatment with 0.5–2% H_2_O_2_. Sections were then treated with 3% goat, horse and/or swine serum (Sigma, St. Louis, MO; DAKO, Inc., Carpinteria, CA) and 0.3% Triton X (TX) in 0.01 M PB, 0.15 M NaCl, pH 7.4 (PBS) for 1 h to reduce nonspecific staining. Following rinses in PBS, sections were incubated for 48–72 h at 4^o^ C in the primary antibodies: mouse monoclonal anti-glial fibrillary acidic protein (GFAP), (DAKO, Inc.), 1:2000 (1:750 for immunofluorescence (IF); rabbit polyclonal anti-S100β (Abcam, Inc., Cambridge, MA), 1:1000; mouse monoclonal anti-myelin basic protein (MBP), (Chemicon International, Inc., Temecula, CA), 1:500; mouse monoclonal anti-MAP 2 (Sigma), 1:2000 (1:1000 for IF); rabbit polyclonal anti-Iba1 (ionized calcium binding adaptor molecule 1; Wako Chemicals USA, Inc., Richmond, VA), 1:2000 (1:1000 for IF); mouse-monoclonal anti-Kv2.1 (kindly provided by Dr. J.S. Trimmer; UC Davis), 1:500 for IF; rabbit polyclonal anti-eGFP (Invitrogen, A11122: 1:1000) and rabbit polyclonal antibody against ubiquitin (DAKO, Inc.), 1:2000, (1:1000 for IF) in PBS containing 1% goat, horse or swine serum, 2% BSA and 0.3% TX. Following rinses in PBS, sections were incubated in biotinylated goat or swine anti-rabbit IgG (DAKO, Inc.; Vector Laboratories, Burlingame, CA), diluted 1:500 for 24 h at 4 °C. After rinses in PBS, sections were incubated in ABC (Elite ABC Kit, Vector Laboratories), diluted 1:500 in 2% BSA, 0.3% TX and PBS for 24 h at 4 °C. After rinses in PB followed by Tris-HCl buffers (pH 7.4; 7.6), sections were incubated in 0.025% 3,3′-diaminobenzidine (DAB, Sigma) with 0.003% H_2_O_2_ in TB (pH 7.6). The incubation was stopped by rinses in TB and PB. For double-immunostaining (e.g., GFAP/ubiquitin-colocalization) differently-colored chromogens were used (DAB; Vector SG Substrate Kits, Vector Laboratories). Specificity of the immunostaining was evaluated by omitting primary antibodies from the regular staining. Sections were mounted on slides, dehydrated, cleared, and cover-slipped with Permount.

### Immunofluorescence staining

For single and multiplex immunofluorescent labeling of ubiquitin and neuronal/glial cell markers, frozen sections were transferred into buffered 30% and/or 10% sucrose, then rinsed in 0.1 M PB and treated with 0.1% sodium borohydride for 15 min. Thereafter, sections were rinsed again with 0.1 M PB and then permeabilized with 0.5% H_2_O_2_ in 0.1 M PB for 15 min followed by rinses in 0.1 M PB and 0.01 M PBS. Free-floating sections were treated with 10% goat serum in 0.01 M PBS containing 0.3% TX-100 (vehicle) for 1 h and then incubated overnight at 4 °C in vehicle containing different combinations of mouse monoclonal/rabbit polyclonal antibodies of different IgG isotypes (see above). After rinses in 0.01 M PBS and 10% goat serum (vehicle), sections were incubated in isotype-specific Alexa-conjugated secondary antibodies (1:2000): Alexa 568- and/or 488-labeled goat anti-rabbit IgG and/or Alexa 488 and/or 568-labeled goat anti-mouse IgG (Invitrogen, Carlsbad, CA) for 1–2 h as described previously [57]. Following rinses in vehicle, sections were mounted on gelatin-coated slides and cover-slipped with mounting medium containing DAPI (4′, 6-diamidino-2-phenyindole di-lactate) for nuclear staining (Vectashield “Hard Set”, Vector Laboratories).

Repeat-associated non-ATG (i.e., RAN translation) translation of a novel potentially toxic peptide, FMRpolyG, was recently described in the brains of CGG KI mice [47]. Therefore we carried out immunostaining for FMRpolyG in order to determine whether this peptide was also present in the inclusions in the Gfa2-CGG99 mice. Whole Gfa2-CGG99 and WT mouse brains were sectioned sagitally and the hemispheres were fixed overnight in 4% paraformaldehyde and embedded in paraffin according to standard protocols. Sections (6 μm) were cut on a rotary microtome and deparaffinized, followed by antigen retrieval using microwave treatment in 0.01 M sodium citrate. Endogenous peroxidase activity was blocked and immunostaining was performed overnight at 4 °C using mouse anti-GFP (Roche 181446011000), rabbit anti-ubiquitin (Dako Z0458; 1:250), or mouse-anti FMRpolyG (8FM) [9]; 1:10) antibodies. Antigen-antibody complexes were visualized by incubation with DAB substrate (Dako) after incubation with Brightvision poly-HRP-linker (Immunologic). Slides were counterstained with haematoxylin and mounted with Entellan.

Multiplex immunofluorescence staining for FMRpolyG, ubiquitin, GFAP and NeuN were carried out in separate brain sections. Sections were blocked for autofluorescence with Sudan Black in 70% ethanol. Primary antibodies included rabbit-anti ubiquitin (DAKO Z0458; 1:50), mouse-anti ubiquitin (Cytoskeleton AUB01-S; 1:200), mouse-anti FMRpolyG (1:10) [9], rabbit anti-GFAP (Sigma G9269; 1:200), and mouse anti-Map 2 (Roche, 1:400). Secondary antibodies included anti-rabbit Fab Alexa 488 (Life technologies A11070; 1:100) and anti-mouse Cy3 (Jackson Immuno research 715–165-150; 1:100). Nuclei were visualized with Hoechst. Analysis was done with a Leica confocal microscope and Leica Application Suite Advanced Fluorescence (LAS AF) software (Leica Microsystems, Buffalo Grove, IL).

### Electron microscopy

Frozen 30 μm brain sections were collected from CGG KI and Gfa2-CGG99 transgenic mice perfused with 4% PB-buffered PFA, post-fixed for 1 h and stored in 30% sucrose at 80 °C. Sets of sections were thawed and postfixed with Karnovsky fixation solution for 1 h. The sections were then postfixed with 1% buffered osmium tetroxide for 1 h, then thoroughly rinsed with PB and dehydrated in a series of ethanol solutions and flat embedded in a mixture of Epon and Araldite between two aclar sheets for 24 h at 70 °C as previously described [55]. Serial ultrathin sections were collected on TEM grids, and stained with uranyl acetate and lead citrate. Sections were then examined in a Philips 120 electron microscope. Electron microscopic images were acquired digitally using a 2 k × 2 k high resolution CCD camera (Gatan, Pleasanton, CA), and post-processed using Photoshop software.

### Cell identification and evaluation of Intranuclear inclusions

The sections were analyzed using a Nikon ECLIPSE E600 microscope with epifluorescence attachment and digital camera. Images were converted to a file format for processing as an Adobe Photoshop document. Images were analyzed to verify the presence of ubiquitin-positive intranuclear inclusions in different cell types identified with various neuronal and glial cell markers in brains of Gfa2-CGG99, Gfa2-CGG11 and WT mice. Cresyl violet and/or H&E-stained sets of brain sections from transgenic and WT mice at different ages were used for comparison and evaluation of gross anatomical differences. The different cell types in the brain were determined based on standard morphological criteria using Nissl cell staining and neuronal and glial cell markers as described previously [23, 56].

Neurons were identified by their size, large round nuclei, single or multiple nucleoli, and their abundant cytoplasm, as well as by using molecular neuronal markers (e.g., NeuN, Kv2.1 channel protein). These descriptions were confirmed using electron microscopy.

Astroglia were identified by their round/ovoid nuclei with light euchromatin, and absence of nucleoli and cytoplasm. In addition, GFAP- and S100β-immunocytochemistry and/or immunofluorescence were used to identify subpopulations of astroglia based on their differing immunoreactivities (i.e, protoplasmic and/or fibrous astroglia) in different brain regions. These descriptions were confirmed using electron microscopy.

Oligodendrocytes (MBP-immunopositive cells) were identified based on their typical appearance with small, round, hyperchromatic nuclei surrounded by thin somatic cytoplasm, and their localization to white and grey matter.

Microglia were identified primarily on cellular morphology obtained from Iba1 immunostaining which displayed small cell bodies with a round nucleus and fine, ramified processes that are characteristic of resting microglia, and were clearly distinguishable from activated (but non-phagocytic) microglia and phagocytotic cells (i.e., brain macrophages). For all cell types, immunocytochemical staining for ubiquitin was used to specifically label intranuclear inclusions in combination with cell-specific markers to identify the cell type (i.e., neuronal and/or non-neuronal cells). Finally, all cell and inclusion types were determined and confirmed based on their ultrastructural appearance and characteristic features using electron microscopy.

### Laser capture/microdissection (LCM)

To evaluate the cellular specificity of Gfa2-CGG99-eGFP expression, single cell laser capture microdissection (LCM) was performed on ubiquitin-immunolabeled cells. Gfa2-(CGG99)-eGFP, CGG knock-in (KI) and WT mice were euthanized by lethal overdose with sodium pentobarbital after which their brains were rapidly removed and immediately frozen in OCT compound (Ted Pella Inc., Redding, CA). Coronal sections were cut at 12 μm on a cryostat (Leica Microsystems Inc., Buffalo Grove, Il). Sections were direct-mounted onto MMI Membrane Slides (Molecular Machines & Industries AG, Switzerland) and dried for 10 min at room temperature. Sections were then rinsed briefly in water, fixed in 70% ethanol for 1 min, and incubated in rabbit polyclonal antibody against ubiquitin (DAKO, Inc.), 1:100 in 0.1 M PB with 5% goat serum for 1 h at room temperature. After 3 brief washes in 0.1 M PB, sections were incubated in Alexa 488-labeled goat anti-rabbit secondary IgG containing 5% goat serum for 1 h at room temperature. Finally, sections were rinsed 3x briefly in 0.1 M PB, counterstained with DAPI in 0.1 M PB for 1 min and dehydrated through a descending series of alcohols. After complete drying, slides were either directly processed for LCM or stored at -80 °C.

LCM was performed on coronal sections using a MMI CellCut Laser Capture Microscope (Molecular Machines & Industries AG, Switzerland). Individual neurons were captured onto isolation caps of specifically designed centrifuge tubes (MMI) and maintained frozen at -80 °C until RNA purification. Approximately 100–200 individual neurons containing ubiquitin-positive inclusions, but negative for eGFP histofluorescence, were captured from 18 tissue sections from the ventromedial hypothalamus and combined into a single PCR tube. In addition, astrocytes expressing eGFP fluorescence and bearing ubiquitin-positive inclusions were laser-captured from 10 neocortical tissue samples from Gfa2-CGG99-eGFP mice, combined and used as a eGFP-positive control. For comparison 100–200 cells from the amygdala of a CGG KI mouse with a CGG168 repeat expansion but no eGFP were isolated and combined as a negative control.

### RNA isolation and amplification

Total RNA was isolated from LCM samples using a Qiagen RNeasy micro kit (Qiagen, Germantown, MD) according to manufacturer’s recommendations. RNA quantity and quality was estimated using a NanoDrop spectrophotometer (ThermoFisher Scientific, Waltham, MA) and Agilent Bioanalyzer (Agilent Techologies Inc., Santa Clara, CA). RNA from each sample was subjected to linear amplification using Nugen Inc. SPIA technology (Nugen Tehnologies Inc., San Carlos, CA). The quantity and quality of resulting amplified cDNA was assessed using a NanoDrop and Bioanalyzer.

### Semi-quantitative real-time PCR

Real-time PCR was performed using an iCycler (Bio-Rad) to measure incorporation of the fluorescent dye SYBR Green I. For each reaction, a master mix of the following was made: 1× PCR buffer (QIAGEN), 400 mM dNTP, 0.5 mM forward (5′-AGTGGAGAGGGTGAAGGTGA) and reverse (5′-GGTAAAAGGACAGGGCCATC) eGFP primers (Operon), 0.01× SYBR Green I (Invitrogen), 1.5 mM MgCl2, 10 nM FITC (Bio-Rad), and 1 U of TaqDNA polymerase (QIAGEN). All PCRs were optimal for the following cycle conditions, 94 °C (15 s), 60 °C (30 s), and 72 °C (30 s), and were run for approximately 40 cycles. After the PCR, a melting-curve analysis was performed to confirm the specificity of the PCR. In addition, samples of the PCRs were subjected to electrophoresis to verify product size and specificity. The relative quantification of RNA targets was performed as follows: The threshold cycle (Ct) at which a gene of interest first rose above background was determined and subtracted from that of the housekeeping gene, β-actin, the PCR for which was performed in a separate reaction tube. This was termed ΔCt. The ΔCt for each reaction was plotted as 2 − ΔCt. Therefore, all values are for RNA expression normalized to β-actin mRNA.

### Statistical analysis

Behavioral data were analyzed using R 2.14.0 language and environment. Data for each variable were examined for normality using the Shapiro-Wilk test and Kolmogorovo-Smirnov test. Normally distributed data were analyzed by Analysis of Covariance (ANCOVA) with body weight as a covariate. If the assumption of normality of distribution was violated, then group comparisons were carried out using nonparametric rank-based ANCOVA with body weight as a covariate. The minimum levels for statistical significance set at *p* < 0.05 for all statistical analyses. Data in figures are means ± standard error of the mean (SEM). Detailed statistical results for behavioral experiments are provided in Additional file 1: Figure S2.

## Results

### Gfa2-CGG99 transgenic mice exhibit neurological and systemic disease phenotypes

#### Body weight

At 6 months of age when behavioral testing began Gfa2-CGG99 mice had significantly lower body weights (31.1 ± 1.3 g) compared to WT (39.5 ± 1.3 g), and this difference remained significant at 7 and 8 months of age (*p* < 0.01) (Additional file 1: Figure S2). Body weight was therefore used as a covariate in statistical analyses. Body length did not differ between Gfa2-CGG99 (93.6 ± 0.6 mm) and WT mice (94.9 ± 0.5 mm) at the start of behavioral testing.

#### Rotarod

**Fig. 2:**
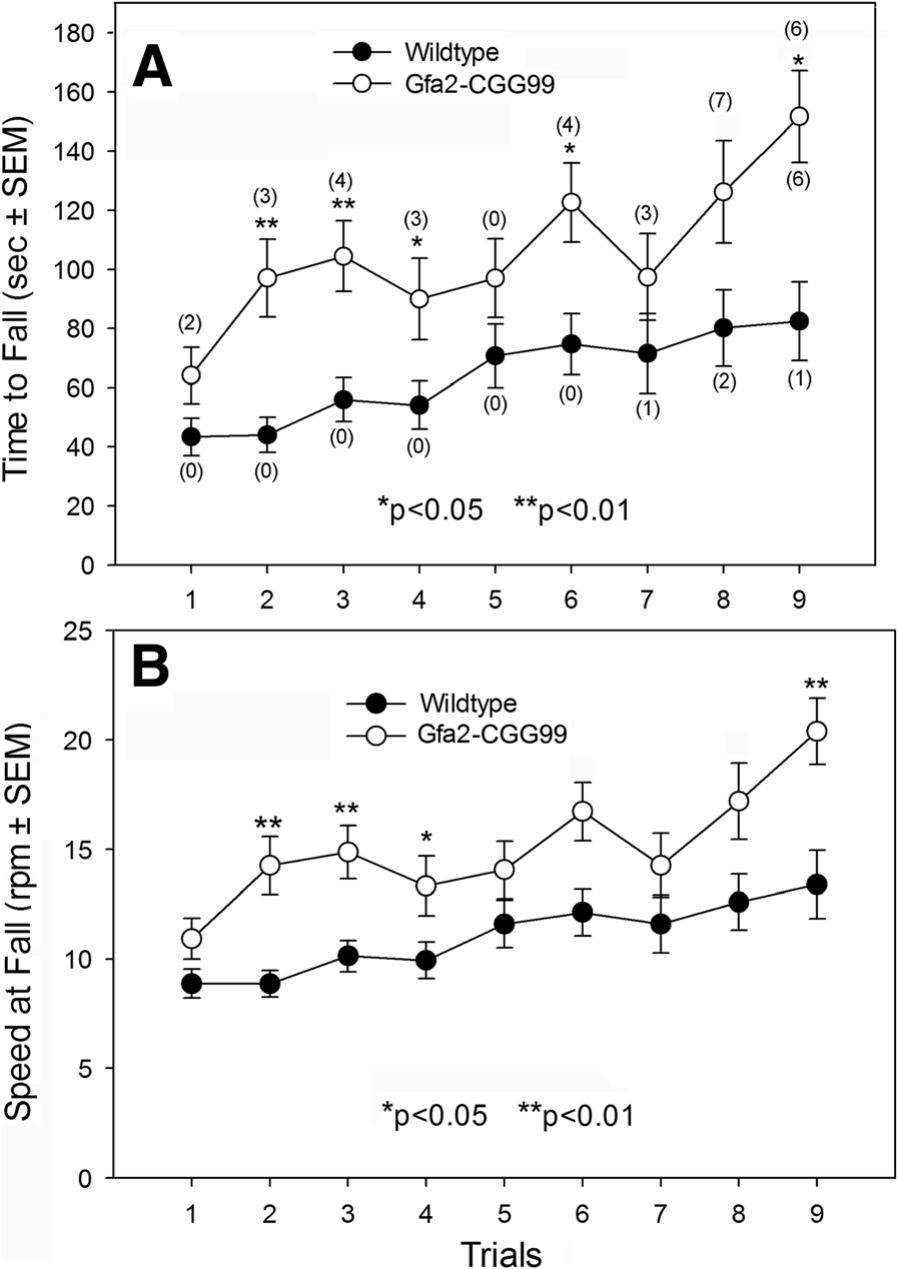
Rotarod performance of Gfa2-CGG99 and WT mice. **a** Time to fall from the rotarod was significantly longer for Gfa2-CGG99 versus WT mice. In addition, Gfa2-CGG99 mice also showed significantly more flips (number of flips shown in parentheses) than WT mice. **b** The speed at which Gfa2-CGG99 mice fell from the rotarod was significantly higher than WT mice. *p < 0.05, **p < 0.01

As shown in Fig. 2, Gfa2-CGG99 mice stayed on the rotarod significantly longer (e.g., A. Time to Fall) than WT mice on trials 2, 3, 4, 6 & 9, but not on trial 1. A similar analysis showed that Speed to Fall (Fig. 2b) was significantly longer for Gfa2 compared to WT on trials 2, 3, 4, & 9 (*p* < 0.05). Numbers in parenthesis in Fig. 2a show that Gfa2-CGG99 mice flipped (i.e., clinging to the rotarod cylinder through complete 360 deg. rotations) on 8 of 9 trials compared to only 3 of 9 trials for WT (*p* < 0.05). Eleven of 15 Gfa2 mice showed one or more episodes of flipping compared to 2 of 15 WT mice (*p* < 0.01).

#### Gait analysis

**Fig. 3:**
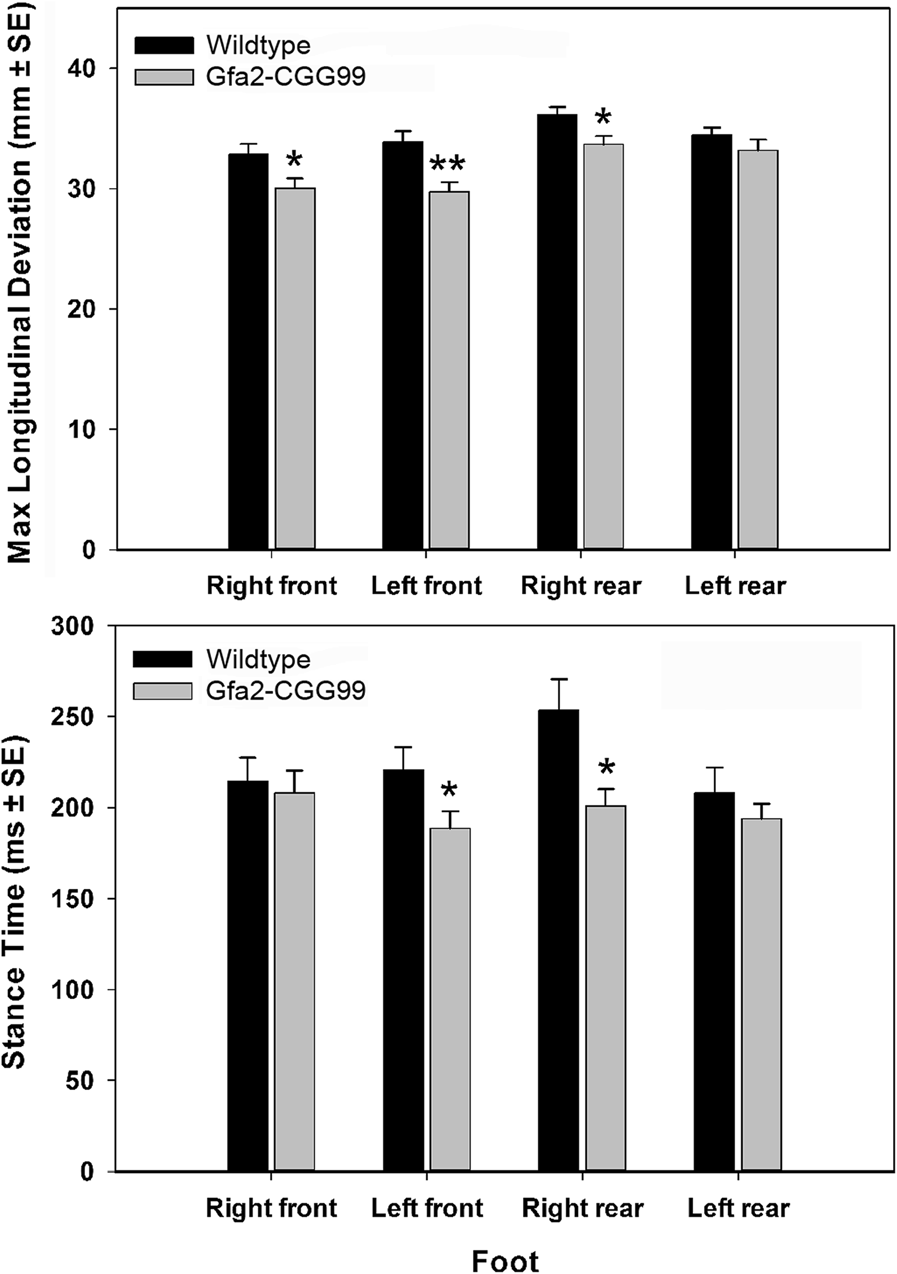
Gait analysis. *A. maximum* longitudinal deviation was significantly shorter for the right and left front and left rear feet of Gfa2-CGG99 mice compared to WT mice. B. Stance time was significantly shorter for Gfa2-CGG99 compared to WT mice for the left front and right rear feet. Mice were tested at 7 months of age; *n* = 15 per group. *p < 0.05, **p < 0.01

Gfa2-CGG99 mice differed from WT mice in several basic gait parameters measured in the TredScan apparatus (Fig. 3). Gfa2-CGG99 mice had shorter stance times (time in contact with floor) for front-left and rear-right feet compared to wild type controls (*p* < 0.05). Maximum longitudinal deviation was significantly shorter in Gfa2-CGG99 mice compared to WT for the front-left (*p* < 0.05), front-right (*p* < 0.05) and rear-right (*p* < 0.05), indicating a shortened range of motion for the Gfa2-CGG99 versus WT mice. No other significant gait differences were found between genotypes for any other measure. When adjusted for body weight differences these gait effects were no longer statistically significant.

#### Ladder rung test

**Fig. 4:**
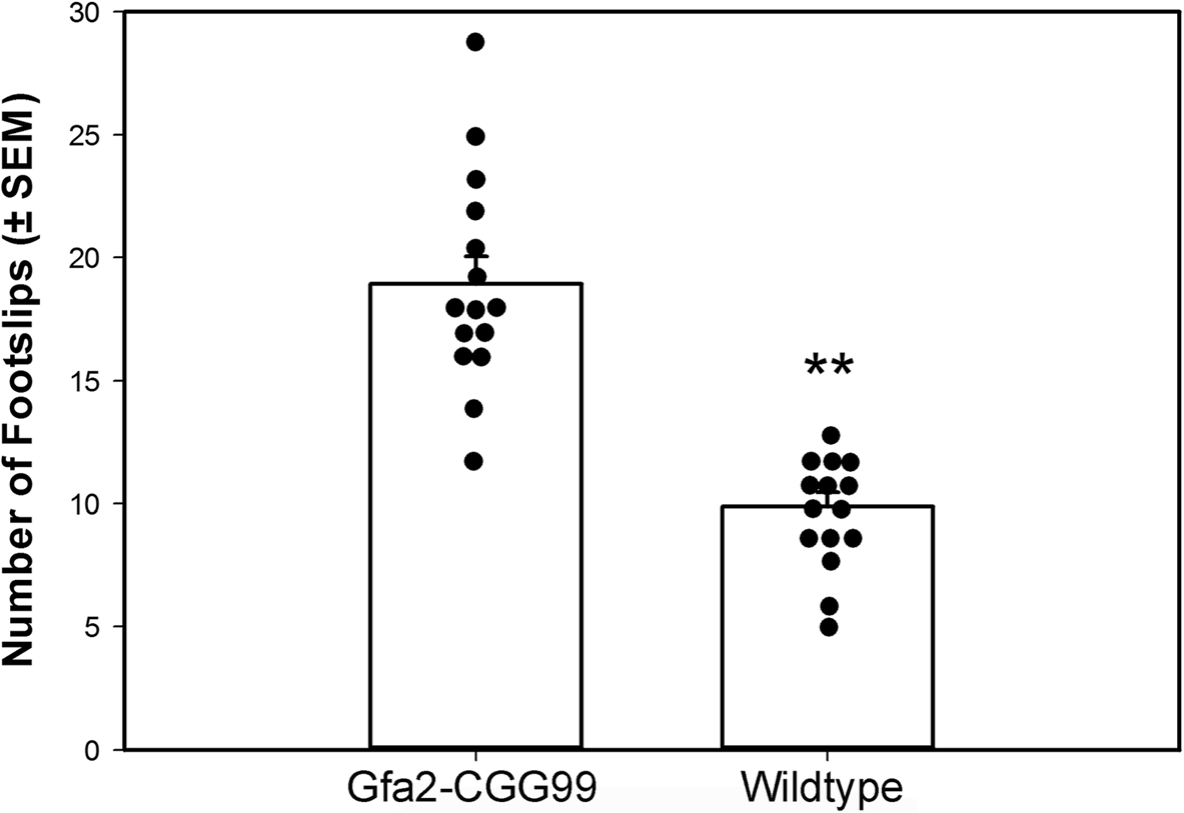
Gfa2-CGG99 mice made significantly more foot slips than WT mice in the ladder rung task. Mice were tested at 8 months of age; *n* = 15 per group. **p < 0.01

Gfa2-CGG99 mice made significantly more foot slips while crossing the ladder run apparatus compared to WT controls (Fig. 4). A one way ANCOVA with body weight and locomotor activity as covariates showed that this difference between groups was statistically significant (*p* < 0.001).

#### Anxiety tests

No statistically significant differences in measures of anxiety were found between Gfa2-CGG99 and WT mice in the elevated plus-maze (time in open arm) or open field tests (margin time). Interestingly, Gfa2-CGG99 mice showed an increased frequency of rearing behaviors compared WT mice (*p* < 0.05).

#### Contextual fear conditioning

No differences were found between WT and Gfa2-CGG99 mice for either contextual or cued fear conditioning.

### Intranuclear inclusions in neurons and astroglia in CGG Knock-in (KI) mice

**Fig. 5:**
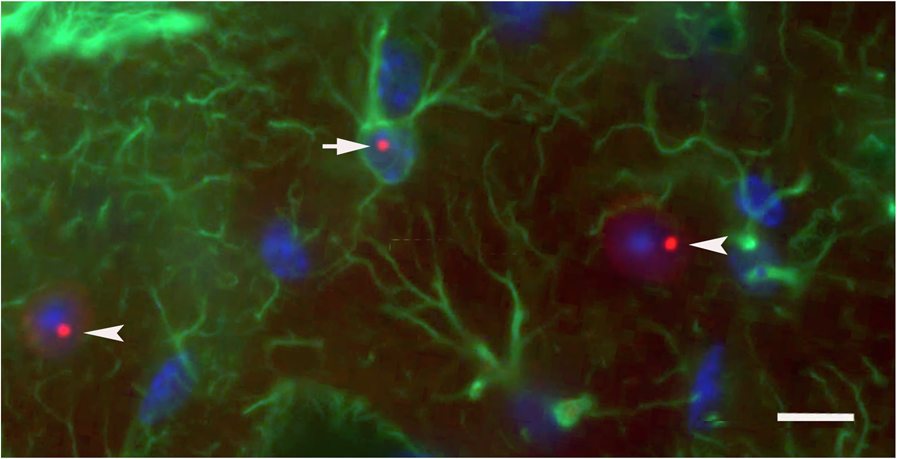
Immunofluorescent labeling and ultrastructure of ubiquitin-positive intranuclear inclusions in neurons and astroglia of the CGG KI mouse. Ubiquitin-positive intranuclear inclusions (red) in a protoplasmic astroglia (green; arrow) and pyramidal neurons (arrow heads) in the neocortex of a CGG128 KI mouse. These fluorescently labeled intranuclear inclusions are shown for comparison with inclusions found in astrocytes in the Gfa2-CGG99 mouse brain. Ubiquitin was immunofluorescently labeled red, GFAP green, and nuclei stained blue using DAPI

Ubiquitin-positive intranuclear inclusions are the hallmark neuropathology in FXTAS patients [26, 27], and similar appearing inclusions are found in a CGG knock-in (KI) mouse model of the fragile X premutation [61, 64]. Figure 5 shows representative red immunofluorescent staining for ubiquitin-positive intranuclear inclusions in neurons (arrowheads) and in a astrocytes (arrow). Astrocyte was labeled immunofluorescent green for GFAP. Brain section is from layer I of the parietal cortex of a 16 month old CGG KI mouse with a 128 CGG trinucleotide repeat expansion. Ubiquitin-positive inclusions were never observed in neurons or astroglia of WT mice used in this study, or in our previous studies in any brain region at any age [46].

### Expression pattern of eGFP in astroglia of Gfa2-CGG99 and Gfa2-CGG11 mice (Fig. 6)

**Fig. 6:**
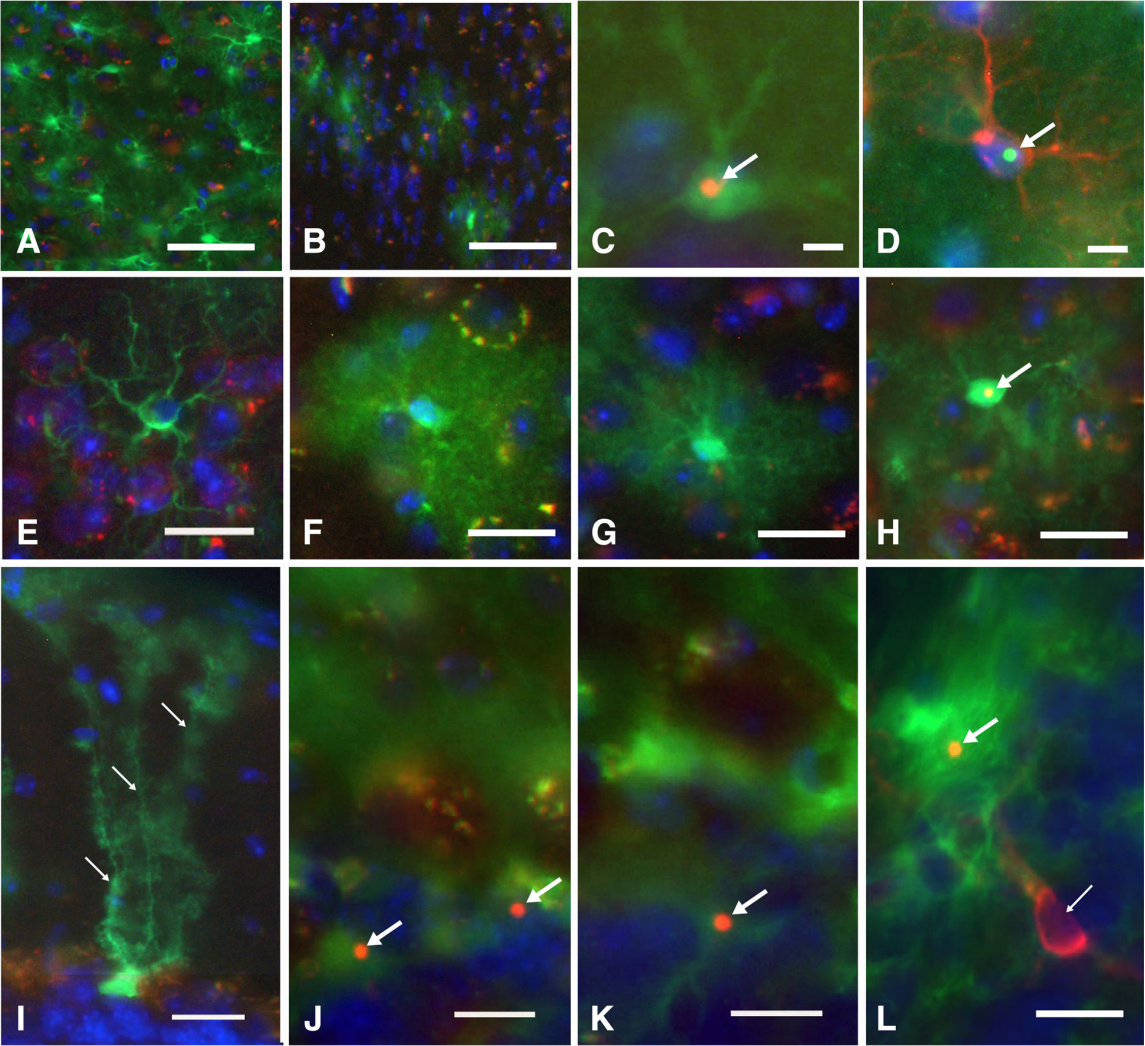
Intranuclear inclusions in neocortical astrocytes and Bergmann glia in Gfa2-CGG99 mice. **a**-**b** EGFP histofluorescence (green) in neocortical astrocytes from a (**a**) Gfa2-CGG99, and a (**b**) Gfa2-CGG11 transgenic mouse. The majority of astrocytes in the Gfa2-CGG99 mouse cortex expressed eGFP, while fewer astrocytes showed expression in the Gfa2-CGG11 mouse. Sections were immunoreacted against ubiquitin (red) and nuclei stained with DAPI (blue). Red/yellow fluorescent puncta are due to autofluorescence of lipofuscin granules in neurons and microglia associated with normal aging in the mouse cortex. Scale bars: 50 μm. **c**-**d** Intranuclear inclusion (**c**) in an eGFP histofluorescent Gfa2-CGG99 mouse astrocyte immunostained for ubiquitin (arrow, orange/yellow). **d** Ubiquitin-stained intranuclear inclusion (arrow, cyan) in a Gfa2-CGG99 astrocyte verified as an astrocyte by staining with anti-GFAP (red fluorescence). Scale bars: 5 μm. **e**-**h** Typically appearing protoplasmic astrocyte from a WT mouse immunostained for Gfap (**e**, green). Shown for comparison with histofluorescent (green) Gfa2-CGG99 astrocytes in **f**-**h**). **f** Green histofluorescence in an astrocyte from a Gfa2-CGG11 transgenic mouse, and from a (**g**) Gfa2-CGG99 transgenic mouse. Both show a cloud-like histofluorescence emanating from their astrocytic processes, and neither contained a ubiquitin-positive intranuclear inclusion. **h** Intranuclear inclusion (arrow, yellow) in a green histofluorescence astrocyte from a Gfa2-CGG99 mouse. Although not quantified, astrocytes in Gfa2-CGG99 mice with inclusions appeared to show less green eGFP histofluorescence compared to astrocytes without inclusions (e.g., compare green histofluorescence in **g** and **h**). Scale bars 10 μm. **I**-**L** EGFP histofluorescence (green) in cerebellar Bergmann glia from a Gfa2-CGG11 (**i**) and Gfa2-CGG99 (**j**) transgenic mouse. In panel, **i** note the green histofluorescence in the soma and radial glial processes of the Bergmann glia in the Gfa2-CGG11 mouse (small arrows). Ubiquitin-stained intranuclear inclusions (arrows, red fluorescence) in Bergmann glia (Figs. **j** and **l**, arrows) and in a protoplasmic astroglia in the granule cell layer of the cerebellum (Fig. 6**k**, arrow) from a Gfa2-CGG99 mouse. Microglia immunolabeled with Iba1 (red fluorescence) did not show eGFP histofluorescence and did not have ubiquitin-stained intranuclear inclusions (**l**, small arrow). Scale bars 10 μm. Figs. **a**-**c**,**e**-**l** immunostained ubiquitin visualized with Alexa 568 2^o^ antibody (red). Fig. **d**, immunostained ubiquitin visualized with Alexa 488 fluorescent 2^o^ antibody (green), astrocytes immunostained for GFAP and visualized with Alexa 568 (red). Fig. **e**, astrocyte immunostained for GFAP and visualized with Alexa 488 (green). Fig. L, microglia immunostained for Iba1 and visualized with Alexa 568 (red). Figs. **a**-**l** nuclei stained with DAPI (blue)

As expected, astrocytes showed green eGFP histofluorescence through the brain in Gfa2-CGG99-eGFP (Fig. 6a) and Gfa2-CGG11-eGFP (Fig. 6b) mice. This is shown for the rostral neocortex where eGFP expression was higher in Gfa2-CGG99 (Fig. 6a) compared to Gfa2-CGG11 mice (Fig. 6b). Gfa2-CGG99 mice showed eGFP histofluorescence in the majority of astroglia across all brain regions (e.g., neocortex, hippocampus, cerebellum, brain stem nuclei). High levels of eGFP histofluorescence were seen in somata, as well as the larger processes and majority of fine processes which appeared as a “green-fluorescent cloud” around the cell body (i.e., see Fig. 6f and g). Immuno-labeling astrocytes with GFAP, microglia with Iba1 and neurons with NeuN or MAP 2 was carried out to determine specificity of expression of eGFP in the different cell types. The results revealed that eGFP histofluorescence was only observed in astrocytes and Bergmann glia, and not in microglia, oligodendroglia or neurons.

### Gfa2-CGG99 mice display nuclear pathology in astroglia

Although greater than 50% of astroglia in the Gfa2-CGG99 mice expressed eGFP, ubiquitin-positive inclusions were only observed in 0.1–0.5% of predominantly protoplasmic astroglia, depending on age and brain region examined. Astrocytes with inclusions, although low in number, were widely distributed throughout the brain including neocortex, cerebellum and occasionally in subcortical brain regions (e.g., hypothalamus and some brain stem nuclei). Figs. 6c-d show astroglia visualized by eGFP histofluorescence (6C) or co-labeled for the glial marker GFAP (6D) with ubiquitin positive intranuclear inclusions (arrows). The inclusions were spherical bodies averaging 1.82 ± 0.29 μm in diameter located within DAPI-stained nuclei of astroglia (Figs. 6c-d). No ubiquitin-positive intranuclear inclusions were found in astrocytes from WT mice (e.g., Fig. 6e) identified by anti-GFAP immunofluorescence, or in the Gfa2-CGG11 transgenic control mice (e.g., Fig. 6f). Examples of a Gfa2-CGG99 neocortical astrocyte lacking or harboring a ubiquitin-positive intranuclear inclusion are shown in Fig. 6g and h, respectively. Interestingly, eGFP expression appeared to be lower in astrocytes from Gfa2-CGG99 mice that contained ubiquitin-positive intranuclear inclusions. For example, compare eGFP histofluorescence in an astrocyte without an inclusion in 6G to an astrocyte with an inclusion in 6H. Developmentally, intranuclear ubiquitin-positive inclusions in astroglia were first observed in neocortex at 4 months of age, but the number was low and only increased slightly at older age (greater than 10 months). We did not find evidence of increased astrocyte proliferation (i.e., gliosis) in the Gfa2-CGG99 or Gfa2-CGG11 mice.

### Gfa2-CGG99 mice exhibit intranuclear inclusions in Bergmann glia

Glia with characteristic Bergmann cell morphology [7, 32, 42, 56, 59] were observed by eGFP histofluorescence throughout the cerebellum in Gfa2-CGG99 and Gfa2-CGG11 mice. Figure 6i shows a representative example of a Bergmann glia cell from a Gfa2-CGG11 transgenic mouse which has the characteristic morphology of Bergman glia (small arrows point to radial processes). No ubiquitin-positive inclusions were found in Bergmann glia from Gfa2-CGG11 mice. In contrast, ubiquitin-positive intranuclear inclusions averaging 1.88 ± 0.36 μm in diameter were observed in somata of Bergmann glia from Gfa2-CGG99 mice (Figs. 6j, k, l, arrows). It is notable that ubiquitin-positive inclusions were more frequently found in Bergmann glia of the cerebellar lobuli 1, 2 and 10, and rarely in the other lobules. All of the ubiquitin-positive inclusions associated with Bergmann glia had defined spherical structures and were located within DAPI-positive nuclei. In addition to Bergmann glia, ubiquitin-positive inclusions were also seen in the velate protoplasmic astroglia of the granule layer, and in astroglia located within the molecular layer and white matter of the cerebellum (not shown). Ubiquitin-positive inclusions were not found in nuclei of Purkinje cells or other cerebellar neurons in Gfa2-CGG99 mice or from Gfa2-CGG11 control mice. We also did not observe a pattern of Purkinje cell loss in Gfa2-CGG99 mice resembling that reported earlier in transgenic mice expressing a similar, but not identical construct [47]. Further, there were no ubiquitin-positive inclusions identified in selectively stained oligodendroglia (not shown) or in microglia (an Iba1-labeled red fluorescent microglia without an inclusion is shown in the bottom right of panel 6 L, small arrow; larger arrow points to an inclusion in an adjacent Bergmann glia).

### Neuronal intranuclear inclusions were found in Gfa2-CGG99 mice

**Fig. 7:**
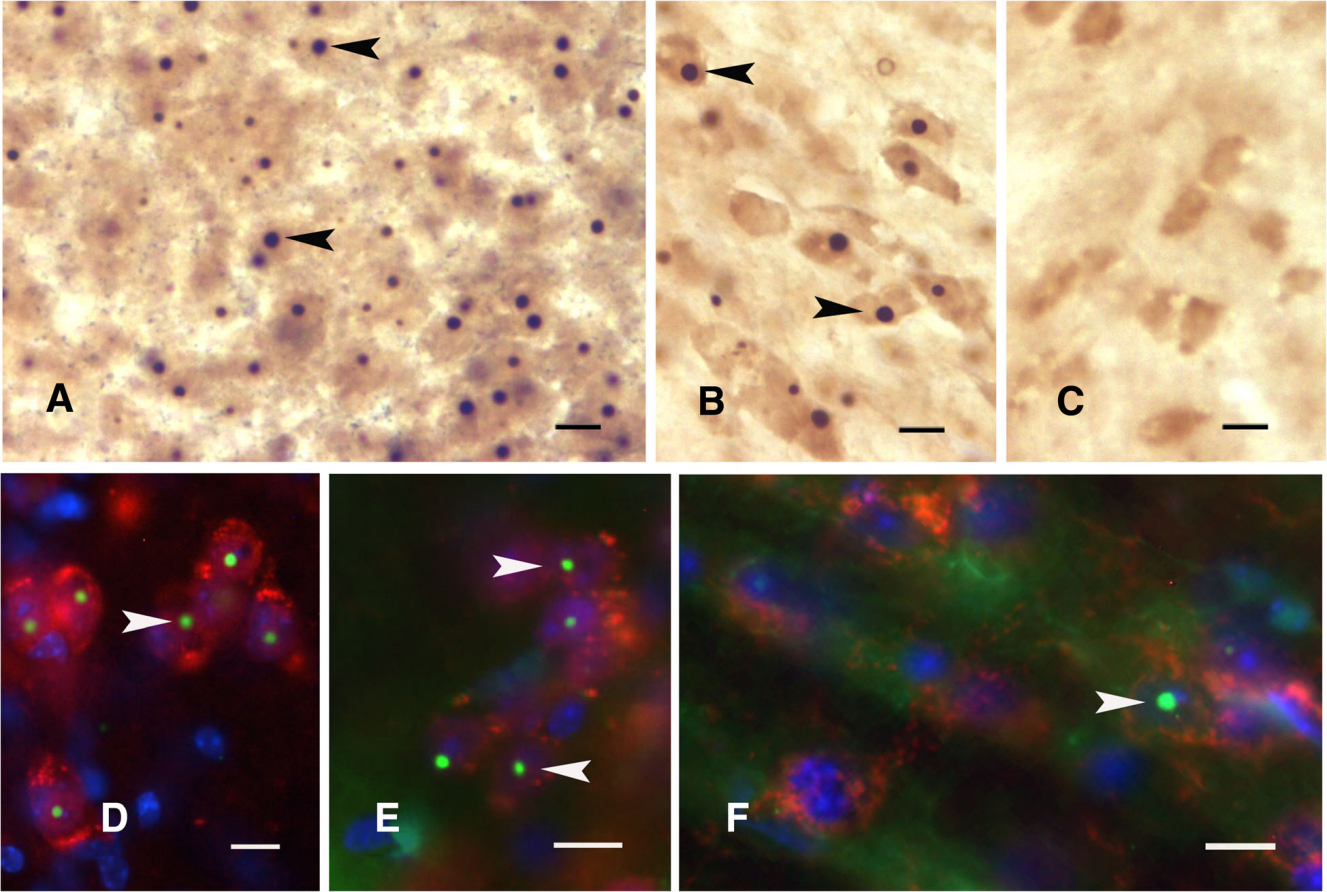
**a**-**c**: Ubiquitin-positive intranuclear inclusions were unexpectedly found in neurons. Neuronal intranuclear inclusions are shown for the paraventricular nucleus (**a**) of the hypothalamus (arrowheads in **a**; brown DAB reaction product) and ventromedial hypothalamus (arrowheads in **b**). No inclusions were found in neurons in Gfa2-CGG11 control mice (**c**) or in WT mice. **d**-**f**: Immunohistofluorescent staining for ubiquitin-positive intranuclear inclusions (arrowhead) in neurons in Gfa2-CGG99 mice in the inferior olivary nucleus (**d**) and suprachiasmatic nucleus (**e**). **f** For comparison, these inclusions are similar to the inclusions found in neurons in the mammillary body of a CGG159 KI mouse. In Figs. **d**-**f**, ubiquitin is immunofluorescently labeled green (Alexa 488, arrowheads) and neurons identified by immunostaining red for NeuN (Alexa 468). Scale bars: **a**-**c**: 5 μm; A-F: 10 μm

Neuronal intranuclear inclusions are key pathological features of CGG KI mice that appear as early as 3 months of age [7, 32, 56, 59]. We did not see and did not expect to see intranuclear inclusions in Gfa2-CGG11 control mice because we were using the Gfa2-specific promoter to limit expression to glia (e.g., Fig. 7c). Unexpectedly, all of the Gfa2-CGG99 mice studied exhibited some ubiquitin-positive inclusions in neuronal nuclei in distinct brain regions, particularly in the hypothalamus, including the paraventricular (Fig. 7a) and ventromedial (Fig. 7b) nuclei. In contrast to these regions, neuronal inclusions in Gfa2-CGG99 mice, although present, occurred less frequently in neocortex and cerebellum. Intranuclear inclusions were particularly large in periventricular nuclei (Fig. 7a). Neuronal intranuclear inclusions were also found in brainstem nuclei (e.g., substantia nigra, reticular formation; data not shown). The appearance of neuronal inclusions in Gfa2-CGG99 mice was age-dependent, with 4–8 month old mice exhibiting only a few neuronal intranuclear inclusions, while older mice displayed higher numbers of inclusions in the brain regions described above. The presence of intranuclear ubiquitin-positive inclusions in neurons was further established by showing co-localization of the inclusions in cells that were identified as neurons by immunofluorescence for well-accepted neuronal markers, including NeuN (Figs. 7d & e, arrowheads), and showing localization within nuclei by DAPI staining. The inclusions showed a similar appearance to those previously described in neurons in the CGG KI mouse (Fig. 7f) [56].

In order to determine whether inclusion formation could be related to “leaky” expression in neurons of the astrocyte-specific Gfa2 promoter in Gfa2-CGG99 mice, brain sections were immunoreacted using multiple antisera for NeuN, GFAP, and/or eGFP. These experiments revealed that (i) ubiquitin-positive inclusions were present in neurons, in addition to astroglia, in various brain regions; (ii) NeuN-positive cells (i.e., neurons) did not show any detectable eGFP histofluorescence and/or GFAP immunoreactivity in the nucleus or cytoplasm; and (iii) only astroglia expressing eGFP (not neurons) were immuno-positive when reacted with antiserum against eGFP and GFAP.

### Absence of eGFP expression in neurons with inclusions analyzed by single cell, laser-capture microdissection (LCM) PCR: Possible evidence for transfer of pathology from astrocytes to neurons

**Fig. 8:**
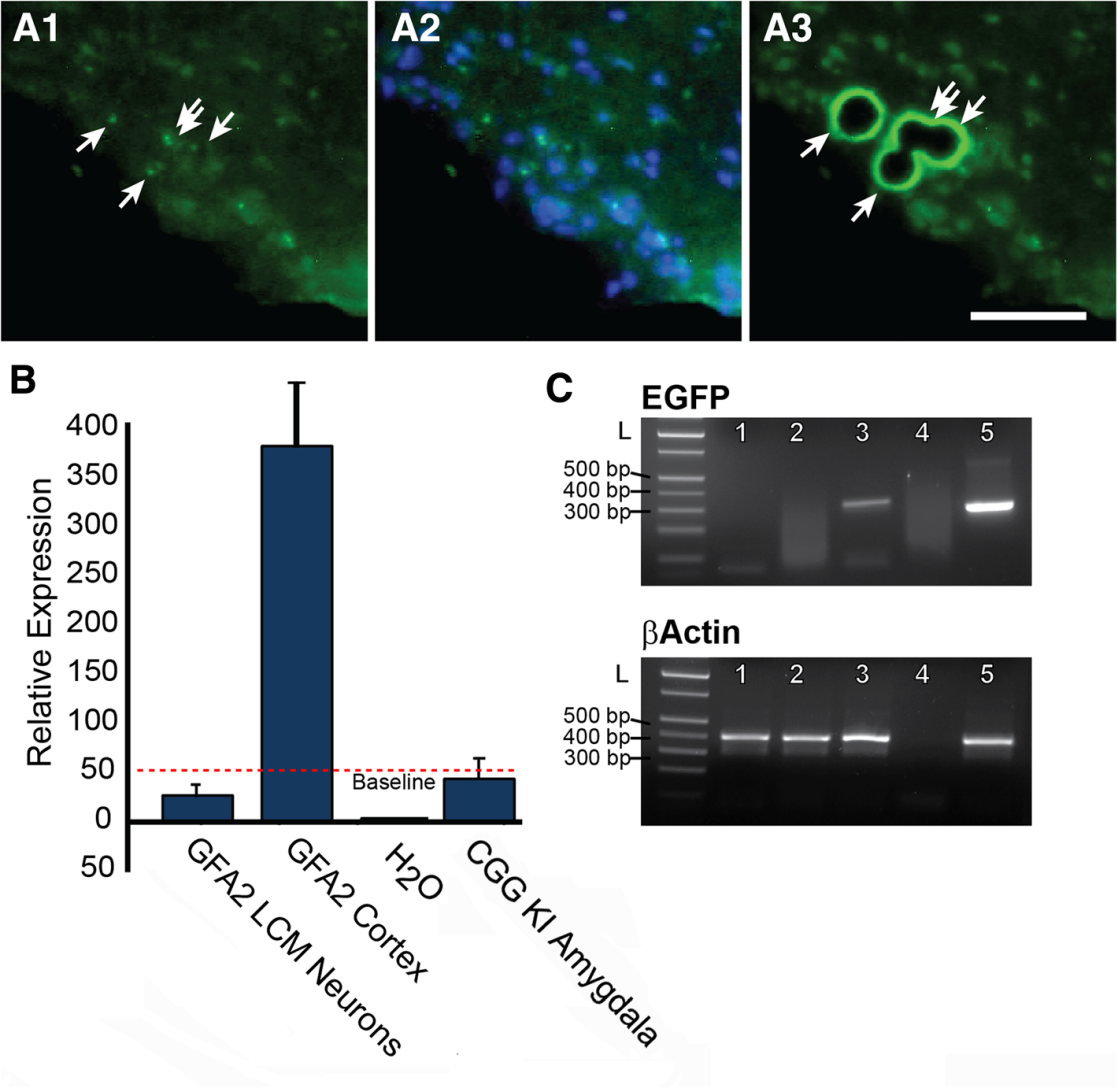
Ventromedial hypothalamic neurons with inclusions from Gfa2-CGG99 mice do not express eGFP by qPCR analysis. A1–3: (**a**1) Neurons in ventromedial hypothalamus (VMH) containing ubiquitin positive inclusions were visualized in 14 μm cryosections of brain by immunofluorescent labeling (green immunofluorescence). (**a**2) Same section with DAPI staining. (A3) Neurons with inclusions were collected by laser capture microdissection (LCM). **b** Total RNA was extracted and amplified from LCM neurons and qPCR analysis was performed to detect expression of eGFP. VMH neurons from Gfa2-CGG99 mice with inclusions did not show detectable expression of eGFP (GFA2 LCM Neurons). In contrast, eGFP expression was readily detected in large LCM samples from cerebral cortex that included astrocytes with eGFP expression (GFA2 Cortex). H_2_O was a water control. As an additional tissue control, single neurons from the amygdala of a CGG KI mouse (168 CGG repeats) were isolated by LCM and analyzed for eGFP expression (CGG KI Amygdala), and as expected since these mice do not carry the eGFP reporter gene, no expression was detected. Red dashed line shows the detection threshold for qPCR analysis. **c** Analysis of qPCR samples by gel electrophoresis confirmed amplification of eGFP in cortical samples containing eGFP positive astrocytes, but not in samples of LCM-isolated hypothalamic neurons. L, DNA ladder; 1, CGG KI amygdala; 2, GFA2 LCM neurons; 3, GFA2 cortex; 4, water; 5, eGFP and/Actin positive plasmid controls. Scale bar = 50 μm

The unexpected finding that neurons without eGFP histofluorescence had inclusions suggested the possibility of cell-to-cell transfer of pathology from astrocytes to neurons in Gfa2-CGG99-eGFP mice. However, it is also possible that eGFP expression in neurons was below the level of detection by histofluorescence. To test this possibility, PCR analysis of laser-capture microdissected cells (LCM) was used to detect possible eGFP mRNA expression in inclusion-bearing neurons in the ventromedial hypothalamus (VMH) of Gfa2-CGG99 mice, a region with numerous neuronal inclusions (e.g., see Fig. 7b). Specifically, neurons in the VMH that lacked eGFP histofluorescence but contained ubiquitin-positive intranuclear inclusions were identified by immunofluoresent staining (Figs. 8a1 & a2), isolated by LCM (Fig. 8a3) and combined for PCR analysis (Fig. 8b & c). Total RNA was isolated, quantified and linearly amplified using NuGEN SPIA technology (www.nugen.com). The resulting cDNA was processed for semi-quantitative real-time PCR with primers specific for EGFP and β-actin. As a positive control, RNA was also isolated from regions of neocortex that included astroglia that expressed eGFP (i.e., GFA2 cortex). In addition, we isolated single cells with inclusions from the amygdala of a CGG168 KI mouse (i.e., CGG KI Amygdala) that should not express eGFP. These neurons express an expanded CGG repeat under the control of an endogenous *Fmr1* promoter, develop neuronal intranuclear inclusions but do not express eGFP [3, 18, 56]. We did not observe expression of eGFP mRNA in VMH neurons with ubiquitin-positive intranuclear inclusions (GFA2 LCM Neurons; Fig. 8b). Similarly, neurons isolated from the amygdala of a CGG168 KI mouse did not express eGFP (Fig. 8b). In contrast, larger cortical samples from Gfa2-CGG99 mice expressed high levels of eGFP mRNA (Fig. 8b) reflecting the presence of astroglia expressing the eGFP protein. The expected size and relative purity of PCR reactions were confirmed by gel electrophoresis (Fig. 8c).

### Intracytoplasmic inclusions in astroglia of Gfa2-CGG99 mice

**Fig. 9:**
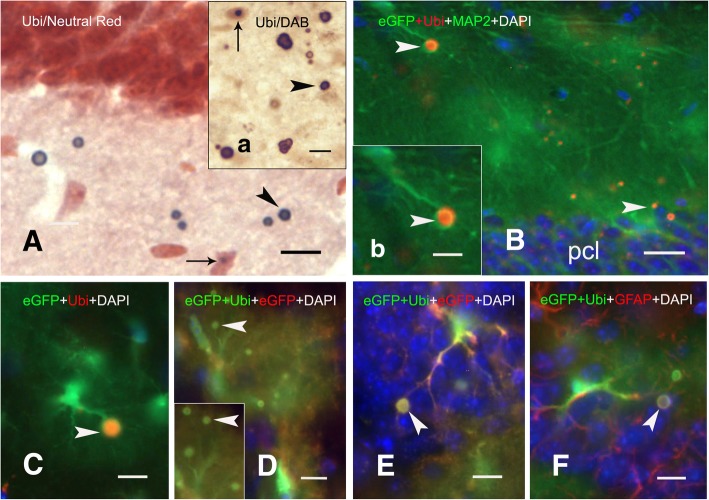
Ectopic and intracytoplasmic ubiquitin-positive inclusions in Gfa2-CGG99-eGFP expressing astroglia. **a** Ubiquitin-positive astrocyte intranuclear inclusion (small arrow) and nearby inclusions that appear to be extracellular (arrowheads) in a neutral-red stained section of hilus from a Gfa2-CGG99 mouse. Insert *a*: Intranuclear (small arrow) and extracellular (arrowhead) ubiquitin-positive inclusions. DAB immuno-peroxidase staining was used to label ubiquitin-positive inclusions in A and inset *a*. **b** Intracytoplasmic ubiquitin-positive inclusion (arrowhead) in an eGFP histofluorescent astrocyte in the stratum oriens of hippocampal CA1 region; pcl pyramidal cell layer. Inset *b* shows higher magnification of the inclusion body (arrowhead). **c** An eGFP histofluorescent astrocyte from a Gfa2-CGG99 mouse with an intracytoplasmic ubiquitin-positive inclusion body (arrowhead) within the hippocampal CA1 stratum radiatum. **d** Ubiquitin-positive intracytoplasmic inclusion (arrowhead, yellowish-green fluorescence) that co-localizes with eGFP in the CA1 pyramidal cell layer (**d**). Inclusions were immunolabeled eGFP (red) and ubiquitin (green), so that co-localization with the inclusions appears as yellowish-green fluorescence. Note that eGFP histofluorescence from expression of the eGFP reporter gene (green) is also present. Nuclei were labeled with DAPI. **d**-**f** Co-localization of intracytoplasmic inclusions (arrowheads) in astrocytes immunofluorescently labeled with ubiquitin (green) and GFAP (red). eGFP histofluorescence from expression of the eGFP reporter gene (green) is also present. Nuclei were labeled with DAPI. Scale bars: **a**-**f**: 10 μm

Ubiquitin-positive inclusion bodies in Gfa2-CGG99 mice did not appear to be restricted to the nuclear compartment (Fig. 9). Initial observations in brain sections from Gfa2-CGG99 mice stained with neutral red and immunostained for ubiquitin revealed inclusion bodies that did not appear to be closely associated with somata of astrocytes or neurons. These ectopic inclusion bodies shown in Fig. 9a were prevalent across grey and white matter of all brain regions examined, but particularly frequent in upper neocortical layers, hippocampus, olfactory bulb and certain brain stem nuclei. These bodies (aggregates) were spherical and averaged 3.62 ± 1.26 μm in diameter. In some regions (e.g., ventromedial hypothalamus) they appeared to form larger clusters of several bodies. They were immunopositive for ubiquitin (dark-blue reaction product) using DAB peroxidase reaction (inset 9a), and/or exhibited ubiquitin immunofluorescence (arrowheads, Fig. 9b-f). Some appeared to be engulfed or surrounded by fine astroglial processes suggesting the inclusion bodies were intracytoplasmic in astroglia (Figs. 9b-c, inset b; arrowheads). Co-localization of inclusions with eGFP (Fig. 9d & e) and GFAP (Fig. 9f) confirmed the association of these inclusions with astrocytes. This observation was confirmed by electron microscopy (EM) as described below (Figs. 11i & k).

### The RAN translation product FMRpolyG is present in inclusions found in the Gfa2-CGG99 mice

**Fig. 10:**
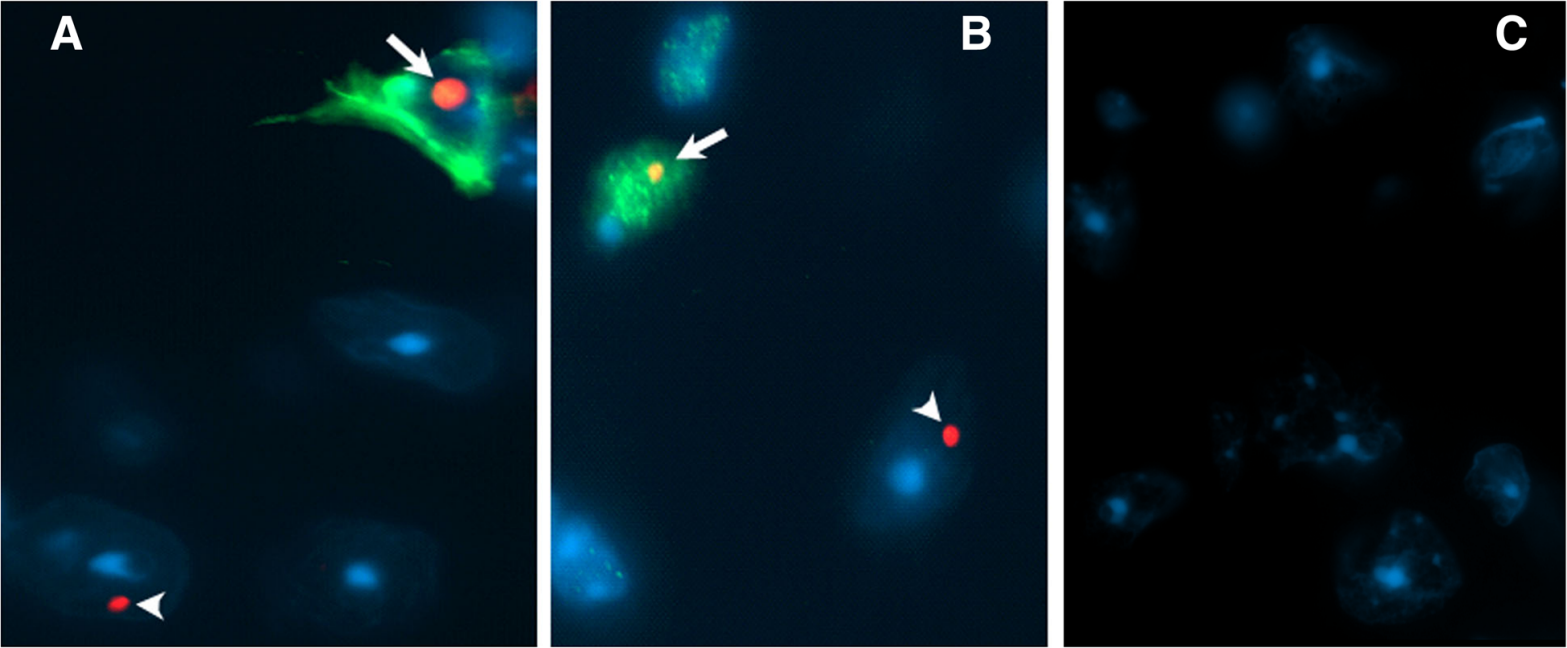
Double immunofluorescent staining reveals FMRpolyG positive inclusion bodies in both astrocytes (**a**) and neurons (**b**) from Gfa2-CGG99 mice. **a**. Photomicrograph showing FMRpolyG-positive inclusion bodies (red) located within GFAP positive astrocytes (green; arrow) as well as in a GFAP negative cell that is probably neuronal (arrow head). **b**. Photomicrograph showing FMRpolyG-positive inclusion bodies (red) located within a MAP 2 positive (green; arrow) neuron as well as in a MAP 2 negative cell that is probably an astrocyte (arrow head). **c** Representative brain section from a Gfa2-CGG99 mouse processed for immunofluorescence but without 8FM mouse anti-FMRpolyG primary antibody

Figure 10a shows an FMRpolyG-positive (red fluorescent) intranuclear inclusion in a GFAP-positive (green) neocortical astroglia from Gfa2-CGG99 mouse (arrow). This observation provides the first evidence for RAN translation in astroglia in a mouse model of the FXTAS. In addition, FMRpolyG immunostaining was also seen in an inclusion body in MAP 2-positive (green) neuron in the hypothalamus (arrow, Fig. 10b). Arrowheads in 10A and 10B, show FMRpolyG-positive inclusions that are likely in an unlabeled neuron and an unlabeled astrocyte, respectively. Combined with the evidence against eGFP transgene expression in neurons (i.e., Fig. 8; laser-microdissection single-cell PCR for eGFP), the finding of FMRpolyG in neurons suggests that some form of cell-to-cell transfer of pathology, possibly involving FMRpolyG, may occur in Gfa2-CGG99 mouse brains. This could be similar to a recent report of cell-to-cell transfer of RAN translation peptides in other models of trinucleotide (or hexanucleotide) repeat disorders [58].

### Electron microscopy (EM) of inclusion bodies in astrocytes and neurons

#### Neurons and astroglia of CGG KI mice)

**Fig. 11:**
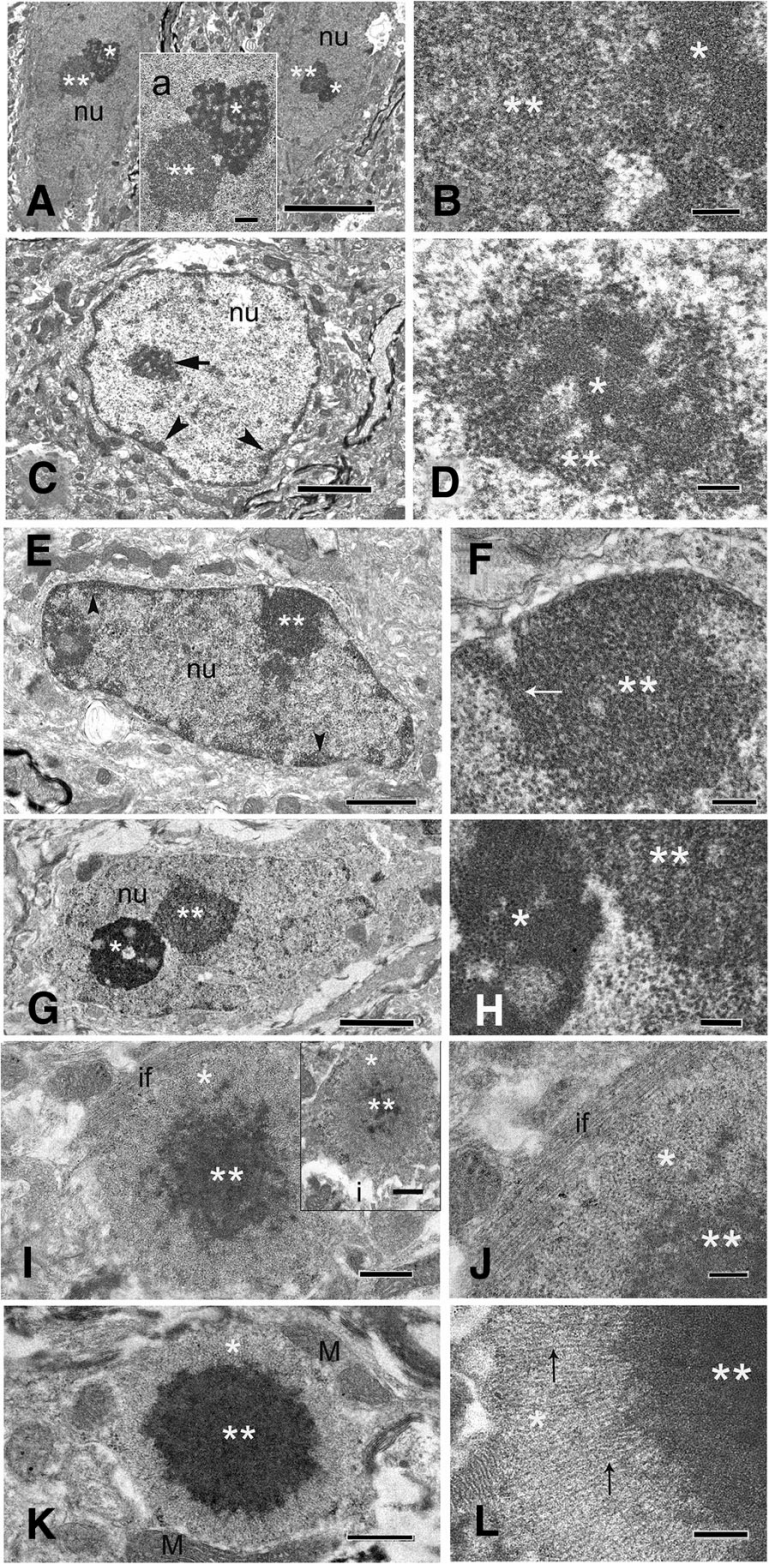
**a** Electron micrographs of two layer 3 pyramidal neurons in the neocortex from a 15.5 month-old CGG159 KI mouse (159 repeats) showing the nucleoli (asterisk) and intranuclear inclusions (double asterisks) in each of the nuclei (nu). Inset “a” shows the nucleolus (asterisk) and a non-membrane bound electron-dense inclusion (double asterisks) of the left neuron. **b** Higher magnification of the adjacent region between the nucleolus (single asterisk) and intranuclear inclusion (double asterisks) from inset “a”. Note the granulo-filamentous ultrastructure of the inclusion material. **c** Electron micrograph of a protoplasmic astroglia in the neocortex of the same CGG KI mouse showing the marginal localization of the heterochromatin (arrowheads) and an intranuclear inclusion (arrow) within the nucleus (nu). **d** The inclusion in C shown at higher magnification consists of a predominantly granular material (double asterisks) surrounding some chromatin-like dense material (single asterisk) within the center. Scale bars: **a**, 10 μm; **b**, 1 μm; **b**, 5 μm; **c**, **e**, 0.2 μm; **d**, 2 μm. **e** Electron micrograph of a fibrous astroglia in the posterior hypothalamus of a Gfa2-CGG99 mouse with an intranuclear inclusion (double asterisk) within the nucleus (nu). Note the marginal chromatin localization (arrowheads) in the nucleus. **f** Higher magnification of the intranuclear inclusion (double asterisk) reveals an electron-dense structure inclusion body made up of predominantly granular material and filaments (small arrow). Scale bars: M 1 μm; N 0.2 μm. **g** Electron micrograph of a principal neuron in the posterior hypothalamus of a Gfa2-CGG99 mouse that shows the nucleolus (single asterisk) and an intranuclear inclusion (double asterisks) within the nucleus (nu). **h** Higher magnification of the adjacent regions of the nucleolus (single asterisk) and the intranuclear inclusion (double asterisk) in **g** which exhibit different ultrastructural features of the granular-filamentous material in the nucleolus versus the inclusion. Note the higher electron density of the nucleolus (pars granulosa and fibrosa) as compared with the more uniform appearing inclusion material. Scale bars: **g**: 2 μm; **h**: 0.2 μm. **i** Electron micrographs of intracytoplasmic inclusion body located within astrocytic processes in the posterior hypothalamus of a Gfa2-CGG99 mouse. The inclusions display an electron-dense core (double asterisks) and a lighter rim (single asterisk) which varied in size between inclusions (compare with inset i). Note the intermediate filaments within the cytoplasm (if) near the inclusion. **j** Higher magnification of the inclusion body in I presents an amorphous to granular material within the core and a granular-filamentous material within the rim. Note the intermediate filaments (if) - a characteristic feature of the astrocytic cytoplasm. **k** Intracytoplasmic inclusion body in the posterior hypothalamus exhibits a large electron-dense core surrounded by a thinner and less dense rim region. Note the mitochondria (M) in the adjacent cytoplasm of the astrocytic process. **l** Higher magnification of a portion from the inclusion body shows a linear-oriented filamentous material in the rim (asterisk) and a dense granular-filamentous material in the outer zone of the core (small arrows). Scale bars: **i**, i, 1 μm; **k**, 1 μm; **j**, **l**, 0.2 μm

Figure 11a and b (higher magnification) show electron micrographs of inclusions in the nucleus of neocortical pyramidal neurons from a CGG159 KI mouse. Figs. 11c & 11d (higher magnification) show inclusions in the nucleus of an astroglia in the neocortex from the same KI mouse. Nuclei of these cells show characteristic ultrastructural features of a nucleolus (single asterisks) in which the partes granulosa and fibrosa appear as clearly separated regions and in which filaments and/or granules are dominant (Peters et al., 1987). In contrast, inclusions in neurons (double asterisks) appear as compact, non-membrane bound arrangements of more loosely packed ribosome-like granules and fine filaments. Inclusions in CGG KI mice range between 1 and 2.5 μm dia, similar to that reported earlier for immunostained neuronal intranuclear inclusions [56]. Intranuclear inclusions were mostly located closely to the nucleolus and varied in size. Occasionally some neurons contained 2 intranuclear inclusions.

#### Inclusions in astrocytes of Gfa2-CGG99 mice

As shown in Fig. 11e and f (higher magnification), intranuclear ubiquitin-positive inclusions in astroglia appeared as a compact collection of densely packed granulo-filamentous material often closely localized to the marginal chromatin of the nucleus.

#### Inclusions in neurons of Gfa2-CGG99 mice

Figured 11G and 11H (higher magnification) show a representative example of a nucleolus (single asterisk) and proximal intranuclear inclusion (double asterisk) in a neuron in the posterior hypothalamus of a Gfa2-CGG99 mouse. The overall appearance of these inclusions was similar to that seen in the CGG KI mouse described above (compare with Fig. 11a). As shown at high magnification in 11H, inclusions in neurons appeared as compact arrangements of non-membrane bound granulo-filamentous material consisting of densely-packed ribosome-like granules and filaments. Intranuclear inclusions were mostly located closely to the nucleolus and varied in size and shape.

#### Cytoplasmic inclusions in astrocytes

Electron microscopic examination revealed that these apparently non-membrane bound inclusion bodies are intracellular and surrounded by cytoplasm of astroglia processes containing mitochondria, ribosomes/polysomes and intermediate filaments, a characteristic feature of astroglia (Figs. 11i,j and k,l). Some cytoplasmic inclusion bodies contained an electron dense central region (core) exhibiting an amorphous to granular character. This core is surrounded by a less dense peripheral region (rim), in which predominantly loosely packed filaments are present, oriented in linear/radial direction. The adjacent cytoplasm often contained bundles of intermediate filaments and mitochondria. The size and morphology of these inclusions is similar in to the intranuclear neuronal inclusions of FXTAS patients observed by EM in human hippocampus [24] and in dorsal root ganglion cells [22].

## Discussion

Solitary intranuclear ubiquitin-positive inclusions in neurons and astroglia are the hallmark neuropathology in FXTAS [23, 24]. CGG KI mice used to model FXTAS have similar intranuclear inclusions in both neurons and astroglia [3, 6, 56]. The present study focused on pathology in astroglia in a transgenic mouse model of FXTAS that selectively expressed a CGG99 trinucleotide repeat expansion fused to eGFP in astroglia and Bergmann glia (the Gfa2-CGG99 mouse). These investigations revealed that: (i) strong eGFP fluorescence in astrocytes and Bergmann glia was widespread throughout the brain with some glial cells exhibiting ubiquitin-positive inclusions; (ii) subsets of neurons located within the brain and brainstem (e.g., hypothalamus, reticular formation, olivary nuclei) also developed intranuclear ubiquitin-positive inclusions; (iii) intracytoplasmic inclusion bodies were observed, predominantly associated with astrocyte processes; (iv) inclusion bodies in astrocytes and neurons immunolabeled for the RAN translation product FMRpolyG; and (v) Gfa2-CGG99 mice showed primarily a motor deficit phenotype. The rationale for developing transgenic Gfa2-CGG99-eGFP mice was to create a mouse model to determine the role of astroglia in overall FXTAS pathology. To this end, the Gfa2-CGG99 mice provide evidence that pathology mainly restricted to astrocytes can contribute to abnormal motor function, as seen on the ladder rung test, analysis of gait and rotarod performance compared to WT controls. However, the fact that intranuclear inclusions were also found in neurons in the Gfa2-CGG99 mice complicates attempts to attribute specific pathology to either astrocytes or neurons.

Behaviorally, the Gfa2-CGG99 mice displayed an abnormal, shortened gait and were impaired in their ability to skillfully walk along a horizontal ladder (i.e., ladder rung task), slipping through the floor more often than WT mice. These findings of a primary motor phenotype in the Gfa2-CGG99 mice resemble the ataxia observed in FXTAS patients. Unexpectedly, Gfa2-CGG99 mice showed enhanced performance on the rotarod compared to WT littermates that did not appear to be due to differences in body weight. While superior rotarod performance could reflect better motor learning, it could also be due to use of an alternate strategy to stay on the rotarod such as flipping, which was prevalent in Gfa2-CGG99 mice. Enhanced performance on the rotarod by transgenic and KO mice has been reported. For example, neurexin-1α deletion [19], conditional knockout of PTEN in cortex and hippocampus [37], overexpression of human mutant α-Synuclein, SynA53T [41], and Neuroligin-3 R451C knock-in mice [11] show enhanced performance on the rotarod compared to WT mice. A recent study reported that neuroligin-3 mutations in mice increase repetitive behaviors through altered striatal circuitry, and that this may manifest as stereotyped behavior on the rotarod resulting in an apparent improvement in performance [45]. Therefore, it is possible that the superior rotarod performance in Gfa2 mice is the result of both better motor learning and the adoption of repetitive behaviors on the rotarod such as flipping, and that abnormal motor functions are part of the phenotype of this novel Gfa2-CGG99 model of the Fragile X premutation.

Gfa2-CGG99 mice showed widespread expression of eGFP in more than 50% of all astroglia in the brain but less than 0.5% of astrocytes showing eGFP fluorescence had ubiquitin-positive inclusions. This is similar to the CGG KI mouse model of FXTAS where relatively few astrocytes develop ubiquitin-positive intranuclear inclusions [56]. In contrast, 10–20% of astrocytes in postmortem brain tissue from FXTAS patients contain intranuclear inclusions, and there are more inclusions in astrocytes than neurons in several brain region [23]. The reasons for these differences in the prevalence of inclusions in astrocytes and neurons, and between mouse models of FXTAS and FXTAS are unknown. One possibility may be differences in activity of the ubiquitin-proteasome system (UPS) leading to the accumulation of aggregated proteins within the ubiquitin-positive intranuclear inclusions. The UPS is critical for intracellular protein degradation and turnover, including clearing cells of misfolded proteins. Moreover, UPS activity has been reported to be lower in neurons compared to astrocytes and to decrease with age [53]. It is also possible that inclusions form more slowly in astroglia than in neurons in mouse brain, when compared to human neurons and astrocytes.

Astroglia are known to play a major role in regulating neuronal growth and synaptic development [12, 13, 44], and also in the progression of neurodegenerative diseases and neurodevelopmental disorders [33, 38, 49]. In the present study, astroglia, including Bergmann glia, that contained intranuclear inclusions appeared to show lower levels of eGFP fluorescence in soma and often an absence of eGFP in astrocyte processes. This finding suggests eGFP expression may be reduced in astroglia bearing ubiquitin-positive inclusions. A possible link between translational efficiency and CGG repeat number in carriers of premutation alleles has been reported [43]. Specifically, *FMR1* mRNA translational efficiency was reduced in FXTAS patients with CGG repeat expansions in the range of 97–195 CGGs, with translational efficiency directly correlated CGG repeat length [43]. Therefore, it is possible that astroglia with inclusions in the Gfa2-CGG99 mouse brain may have reduced translational efficiency resulting in reduced expression of eGFP. Alternatively, eGFP mRNA or protein may be sequestered by the inclusions, thereby reducing eGFP fluorescence, similar to the sequestration of several other proteins found to be associated with inclusions in FXTAS [34, 48].

GFAP, the major intermediate filament protein, is almost exclusively expressed in astroglia, and is therefore the preferred astrocyte marker in clinical and basic research studies [17, 40]. Use of the GFAP promoter in the present study was therefore expected to limit transgene expression to astroglia. Previous reports have indicated that some portions of both the human and murine promoter may also direct expression of some genes in neurons, but this occurred only in a few instances and not for green fluorescent protein (GFP) [50]. Because intranuclear inclusions were prevalent in distinct neuronal populations, particularly in the hypothalamus, we carried out a careful investigation of the specificity of eGFP/GFAP expression in neurons with intranuclear inclusions. Using LCM-PCR and immunofluorescent staining we failed to find any evidence for expression of eGFP or GFAP in neurons with inclusions. In addition, we did not find expression of eGFP in microglia or oligodendroglia, and this is consistent with a large number of studies using the GFAP promoter [50].

The finding that some neurons in Gfa2-CGG99 mice also develop intranuclear inclusions but do not express GFP opens the possibility that some form of cell-to-cell transfer of pathology from astrocytes to neurons may be occurring. One possibility is that either an RNA transcript or a translational product (e.g. FMRpolyG) is transferred from astrocytes to adjacent neurons. This could explain why inclusions in both astrocytes and neurons stain for FMRpolyG. We do not yet have direct evidence for such a mechanism in Gfa2-CGG99 mice or in carriers of the Fragile X Premutation or in FXTAS. However, cell-to-cell transmission of dipeptide repeat proteins linked to translation of hexanucleotide repeat expansions in ALS and FTD has been reported in vitro in several CNS cell types, including induced pluripotent stem cells from C9orf72-ALS patients. Importantly, transmission was bidirectional, both from astrocytes to neurons and from neurons to astrocytes [58]. Cell-to-cell transfer processes have been reported in Alzheimer’s pathology, Parkinson’s disease, and polyglutamine disease among others [16, 21, 39, 58].

Neurodegenerative diseases have been shown to exhibit various forms of glial-neuronal miscommunication in what has been called non-cell autonomous pathology [21]. While cell-to-cell transfer of pathology may itself be a form of non-cell autonomous pathology, it is also possible that some other process associated with such non-cell autonomous pathology may play a role in the development of neuronal inclusions that are both ubiquitin and FMRpolyG positive. For example, it is known that astrocytes play a critical role in regulating extracellular neurotransmitter levels in the central nervous system, including specific transport mechanisms for glutamate and metabolic pathways for GABA [14]. Non-cell autonomous pathology occurs in the polyglutamine repeat disease Spinocerebellar ataxia type 7 (SCA7), where extensive pathology in Bergmann glia that help maintain extracellular glutamate homeostasis results in glutamate toxicity and subsequent neurodegeneration of cerebellar Purkinje cells [15].

The finding that inclusions in both astrocytes and neurons showed immunofluorescent labeling for FMRpolyG supports the occurrence of repeat-associated non-ATG (RAN) translation in Gfa2-CGG99 mice. RAN translation of a potentially toxic polyglycine-containing peptide, FMRpolyG, has been previously reported in mouse models of the fragile X premutation and in FXTAS postmortem tissue [9, 47, 52]. This peptide is translated from the expanded CGG repeat initiated at a non-canonical ACG codon approximately 35 nucleotides upstream of the start of the expanded CGG repeat segment [47]. The mechanism of toxicity of FMRpolyG may involve disruption of the nuclear lamina of cells, and evidence suggests that it is the carboxy-terminus of FMRpolyG that mediates this toxicity [47]. RAN translation was originally described for CAG/CTG repeat expansions within the coding region of the *ATXN8/ATXN8PS* gene associated with the neurodegenerative disorder spinocerebellar ataxia type 8 (SCA8) [60]. RAN translation appears to be a pathological mechanism in the hexanucleotide repeat expansion disorder C9orf72, the most common genetic mutation associated with ALS-FTD [58], and may also occur in the CAG repeat expansion disorder Huntington’s disease [1]. Our results provide the first evidence that RAN translation, and possible associated pathology, also occurs in astroglia and Bergmann glia.

We performed extensive ultrastructural analysis of the intranuclear inclusions found in astrocyte soma, neurons, and the cytoplasm of astrocytes. We found that the neuronal inclusions differ somewhat from those in astrocytes, appearing as compact collections of non-membrane bound granulo-filamentous material often closely localized to the marginal chromatin of the nucleus. Neuronal inclusions also appeared to be composed of granulo-filamentous material but tended to be located proximal to the nucleolus. In contrast, EM confirmed that intracytoplasmic inclusions were intracellular within astrocytic processes, with some having an electron dense central core with a less dense peripheral rim. These inclusions are similar to those described earlier in neurons of the human hippocampus in postmortem tissue from FXTAS patients [24], and in dorsal root ganglion cells [22]. Whether or not inclusions are toxic, or how they contribute to neuropathology is currently unknown, but their association with reduced eGFP expression in astrocytes and potential cell-to-cell spreading from astrocytes to neurons suggest a role in FXTAS disease pathogenesis.

## Conclusions

Transgenic mice with high expression levels of an expanded CGG-99 trinucleotide repeat driven by a human Gfa2 promoter were developed to examine pathology in glia associated with the Fragile X premutation. Expression in glia was widespread throughout the brain, as visualized by the eGFP reporter expression within the Gfa2-CGG99-eGFP construct. eGFP fluorescence was limited to astroglia and Bergmann glia only, and a subset of these glia also developed ubiquitin-positive intranuclear inclusions between the ages of 4–16 months. Expression of eGFP was not observed in microglia immunolabeled with Iba1 or oligodendroglia immunolabeled with MBP, and intranuclear inclusions were never observed in these glial subtypes. Although we do not yet have direct evidence for cell-to-cell spread of pathology, the unexpected finding of intranuclear inclusions in NeuN-labelled neurons, particularly in the hypothalamus, opens the possibility for this type of transfer of pathology in our Gfa2-CGG99 mouse model of FXTAS. The presence of the RAN translation product FMRpolyG in the astrocyte inclusions indicates that this mechanism of pathology in trinucleotide repeat expansion disease may not be limited to neurons, and may occur in astrocytes in FXTAS patients, though this is yet to be documented. Taken together, our results highlight that FXTAS pathology is complex involving both astrocytes and neurons and their possible interactions. Our findings thus provide important new insights that should be considered when developing therapies for FXTAS in human patients.

## Additional files

Additional file 1: **Figure S1.** Expression vector maps used to generate the (A) EGFP-CGG99-EGFP or (B) EGFP-CGG11-EGFP transgenic mouse lines. **Figure S2.** Statistical results for behavioral experiments. (DOCX 339 kb)
