# Supplementary material for: Astroglial-targeted expression of the fragile X CGG repeat premutation in mice yields RAN translation, motor deficits and possible evidence for cell-to-cell propagation of FXTAS pathology
Source: Acta Neuropathol Commun. 2019 Feb 26;7:27. doi: 10.1186/s40478-019-0677-7 (PMC6390634; doi:10.1186/s40478-019-0677-7)
Supplement: Supplementary file 1 — Figure S1. Expression vector maps used to generate the (A) EGFP-CGG99-EGFP or (B) EGFP-CGG11-EGFP transgenic mouse lines. Figure S2. Statistical results for behavioral experiments. (DOCX 339 kb) [file 40478_2019_677_MOESM1_ESM.docx]

**Electronic Supplementary Material**

**Astroglial-targeted expression of the Fragile X CGG repeat premutation in**

**mice yields RAN translation, motor deficits and possible evidence for cell-to-cell propagation of FXTAS pathology**

**Authors**

H. Jürgen Wenzel^1^, Karl D. Murray^2^, Saif N. Haify^4^, Michael R. Hunsaker^3^,

Jared J. Schwartzer^1^, Kyoungmi Kim^6^, Albert R. La Spada^7^, Bryce L. Sopher^8^

Paul J. Hagerman^5^, Christopher Raske^5^, Lies-Anne Severijnen^4^, Rob Willemsen^4^, Renate K. Hukema^4^, Robert F. Berman^1^

**Author Affiliations**

1 Dept. of Neurological Surgery, University of California, Davis; Davis, CA, USA

2 Dept. of Psychiatry and Behavioral Sciences, University of California, Davis;

Davis, CA, USA

3 Graduate Program in Neuroscience, Univ. of California, Davis; Davis, CA, USA

4 Dept. of Clinical Genetics, Erasmus MC; Rotterdam, The Netherlands.

5 Dept. of Biochemistry and Molecular Medicine, Univ. of California, Davis; Davis, CA, USA

6 Division of Biostatistics, Dept. of Public Health Sciences, Univ. California Davis, Davis, CA USA

7 Depts. of Neurology, Neurobiology, and Cell Biology, and the Duke Center for Neurodegeneration &

Neurotherapeutics, Duke University School of Medicine, Durham, NC, USA

8 Dept. of Neurology, University of Washington School of Medicine, Seattle, WA.

**Corresponding author**

Robert F. Berman, Ph.D., Dept. Neurological Surgery, 1515 Newton Court, University of California Davis, Davis, CA 95618.

E-mail: [rfberman@ucdavis.edu](mailto:rfberman@ucdavis.edu); Phone: (530) 754-5102, Fax: 530-754-5125

**Contents**

1. Expression Vector Maps

2. Statistical results for behavioral experiments

**Additional file 1: Figure S1:** Expression vector maps used to generate the (A) EGFP-CGG99-EGFP or (B) EGFP-CGG11-EGFP transgenic mouse lines. \

*

*

**Additional file 1: Figure S2:** Statistical results for behavioral experiments:


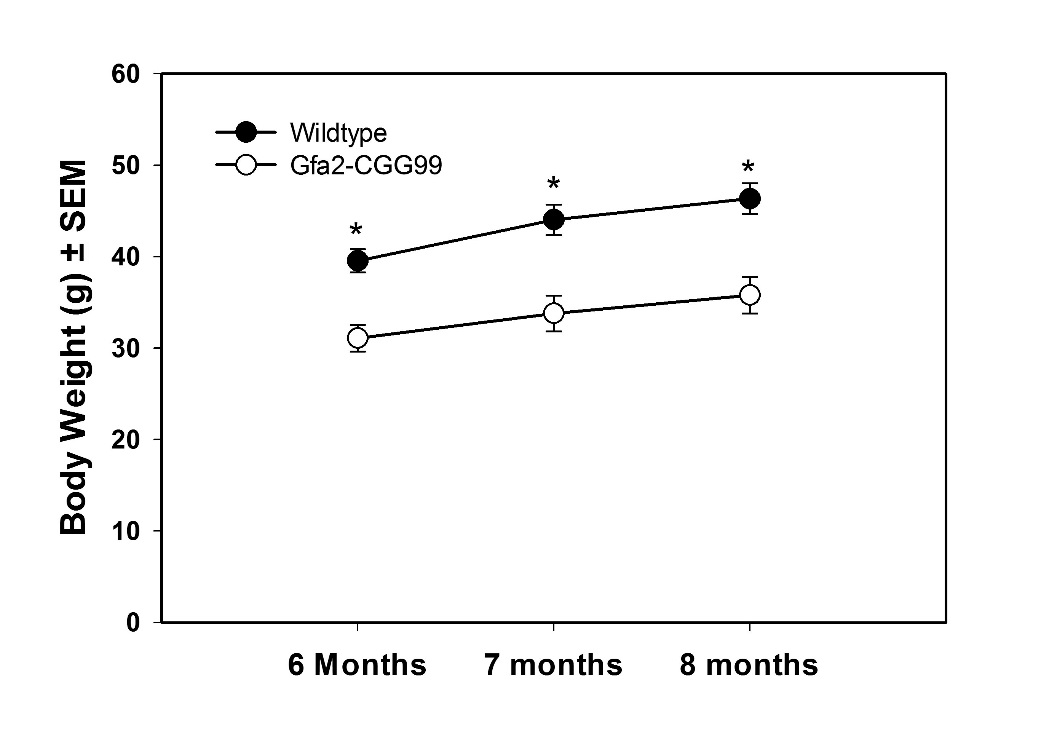
***Body weight:*** Gfa2-CGG99 mice had lower body weights at 6 months of age when behavioral testing began, as well as at 7 and 8 months of age. A repeated measures ANOVA (genotype by age) showed the weight difference between genotypes to be significant [F_(1,18)_ = 18.1, p<0.001], that both groups gained weight over time [F_(2,56)_=50.2, p<0.0010], but that the genotype × age interaction was not significant [F_(2,56)_ =1.96, p=0.15]. Therefore, body weight was used as a covariate in statistical analyses. Body length did not differ between Gfa2-CGG99 (93.6 ± 0.6mm) and WT mice (94.9 ± 0.5mm) [t(28)=1.76, p=0.09] at the start of behavioral testing.

***Rotarod (Figure 2 in manuscript):*** Gfa2-CGG99 mice stayed on the rotarod longer (e.g., A. Time to Fall) and at a higher speed (B. Speed at Fall) compared to WT mice. A repeated measures ANOVA for Time to Fall across trials (genotype X trial) revealed a significant difference between genotypes (F_(1,27)_=6.35, P<0.05) and across trials (F_(8,216)_=2.61, p<0.01). Planned comparisons of WT versus Gfa2-CGG99 across trials showed that differences were statistically significant on days 2, 3, 4, 6 & 9, but the difference the difference between groups on trial 1 was not statistically significant. A similar analysis for Speed at Fall showed a significant genotype × trial interaction (F_(8,21)_=2.118, p<0.05), significant main effect for genotype (F_(1,27)_=8.53, P<0.01) and for trials (F_(8,216)_=2.269, p<0.05). Speed to Fall was significantly longer for Gfa2 compared to WT on trials 2, 3, 4, & 9. In addition, numbers in parenthesis in Figure 2A show that Gfa2-CGG99 mice flipped (i.e., clinging to the rotarod cylinder through complete 360 deg rotations) on 8 of 9 trials compared to only 3 of 9 trials for WT (χ ^2^_(1)_=5.84, p<0.05). Eleven of 15 Gfa2 mice showed one or more episodes of flipping compared to 2 of 15 WT mice (χ ^2^_(1)_=10.9, p<0.01).

***Gait analysis (Figure 3):*** To determine whether Gfa2-CGG99 mice differed in basic gait parameters compared to wild-type controls, treadscan data were analyzed for stance, swing and stride times, as well as for average running speed, right and left foot base and track width, coordination between feet, body rotation and range of motion for each foot (i.e., longitudinal and lateral deviations). Analyses were carried out using a mixed-effect model with foot as within-subject and genotype as between-subject factors. For stance time, a measure of time each foot is in contact with the runway, a significant main effect was found for foot [F_(3, 84)_= 3.99, p<0.01] as well as a significant foot × genotype interaction [F_(3, 84)_= 3.16, p<0.05]. Post hoc analysis revealed Gfa2-CGG99 mice had shorter stance times for front-left [t(28) = 2.09, p < 0.05] and rear-right [t(28) = 2.73, p < 0.05] feet compared to WT controls. No significant differences were found for stride time or swing time between groups. For maximum longitudinal deviation which reflects range of motion, a significant main effect was found for genotype [F_(1,28)_=7.94, p<0.01] as well as a significant foot × genotype interaction [F(3,84)=3.18, p<0.05]. Individual comparisons for each foot showed that the maximum longitudinal deviation was significantly shorter in Gfa2-CGG99 mice for the front-left [t(28)=3.42, p< 0.01], front-right [t(28)=2.33, p< 0.05] and rear-right [t(28)=2.66, p < 0.05], indicating a shortened range of motion for the Gfa2-CGG99 versus WT mice. No other significant differences were found between genotypes for any other measures, including stride-time, stance-time, swing-time, overall stride-length, front or rear stance-width, running-speed and step-angle. When adjusted for body weight differences these gait effects were not longer statistically significant.

***Ladder rung test (Figure 4):*** Gfa2-CGG99 mice made significantly more foot slips while crossing the ladder run apparatus compared to WT controls. A one way ANCOVA with body weight and locomotor activity as covariates showed that this difference between groups was statistically significant [F_(1,26)_=27.6, p<0.001].

***Elevated plus maze:*** For measures of anxiety, no differences were found for the number of entries [open arms: t(28) = 0.88; closed arms: t(28) = 0.70], latency to first enter [open arms: t(28) = 1.69; closed arms: t(28) = 1.20] or total time spent in the open [t(28) = 0.05] and closed [t(28) = 0.80] chambers of the elevated plus maze (p > 0.05 for all measures). Similarly, in the open-field locomotor task, Gfa2-CGG99 mice did not differ from WT mice in the total time spent in the center [t(28) = 1.24], time spent in the margin [t(28) = 1.24] and total number of entries into the center [t(28) = 0.69, p>0.2 for all measures). Interestingly, Gfa2-CGG99 mice showed an increased frequency of rearing behavior, defined as entries into the vertical plane [t(28) = 2.12, p < 0.05] compared to WT mice.

***Contextual fear conditioning (Figure 5):***  No differences were found between WT and Gfa2-CGG99 mice for either contextual [t(28) = 0.12, p>0.9] or cued fear conditioning [t(28) = 0.52, p>0.6].
